# Protocol for generating in-frame seamless knockins in *Drosophila* using the SEED/Harvest technology

**DOI:** 10.1016/j.xpro.2024.102932

**Published:** 2024-07-11

**Authors:** Gustavo Aguilar, Milena Bauer, M. Alessandra Vigano, Isabel Guerrero, Markus Affolter

**Affiliations:** 1 Growth & Development, Biozentrum, University of Basel, Spitalstrasse 41, 4056 Basel, Switzerland; 2 Tissue and Organ Homeostasis, CBMSO (CSIC-UAM), Nicolás Cabrera 1, Madrid, Spain

**Keywords:** Developmental biology, Genetics, CRISPR

## Abstract

The generation of knockins is fundamental to dissect biological systems. SEED/Harvest, a technology based on CRISPR-Cas9, offers a powerful approach for seamless genome editing in *Drosophila*. Here, we present a protocol to tag any gene in the *Drosophila* genome using SEED/Harvest technology. We describe knockin design, plasmid preparation, injection, and insertion screening. We then detail procedures for germline harvesting. The technique combines straightforward cloning and robust screening of insertions, while still resulting in scarless gene editing.

For complete details on the use and execution of this protocol, please refer to Aguilar et al.^1^

## Highlights

• Guide for in-frame knockin design, protein tag selection, and locus analysis
• Step-by-step protocol for the cloning of gRNAs and SEED donors
• Instructions for injecting *Drosophila* embryos
• Guidance on screening, harvesting, and confirmation of knockins

## Abstract (editor highlights)

Publisher’s note: Undertaking any experimental protocol requires adherence to local institutional guidelines for laboratory safety and ethics.

## Graphical abstract

**Figure:**
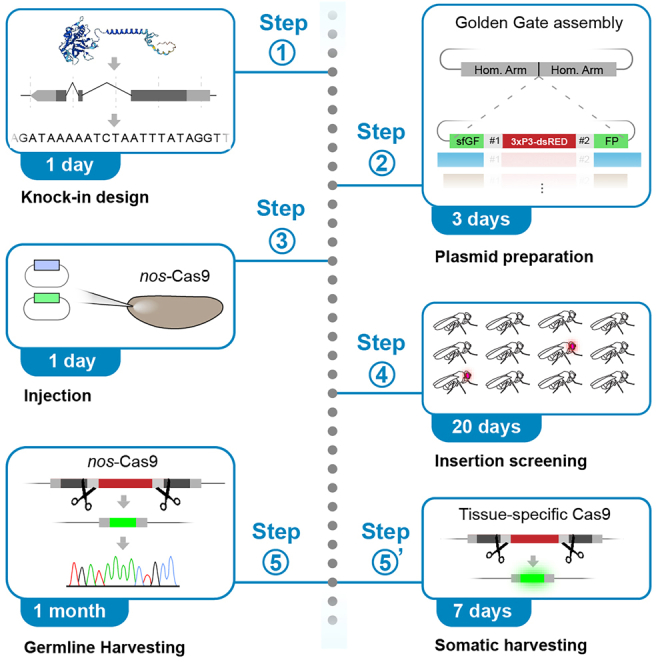

## Before you begin

The precise, targeted manipulation of genetic information is fundamental in biological research. Gene editing has permitted not only the functional dissection of transcriptional and non-transcriptional units, but also the predictable manipulation of protein-coding sequences. When engineering proteins, two manipulations stand out: peptide tagging and the generation of specific allelic variants. The addition of tags is an important step for many downstream applications, such as protein visualization or purification, and more recently, direct protein manipulation *in vivo*. Here we describe in detail the generation of knockins using the SEED/Harvest technology, from donor template generation to the efficient excision of the selectable marker.

### Overview of the technique

**Figure 1:**
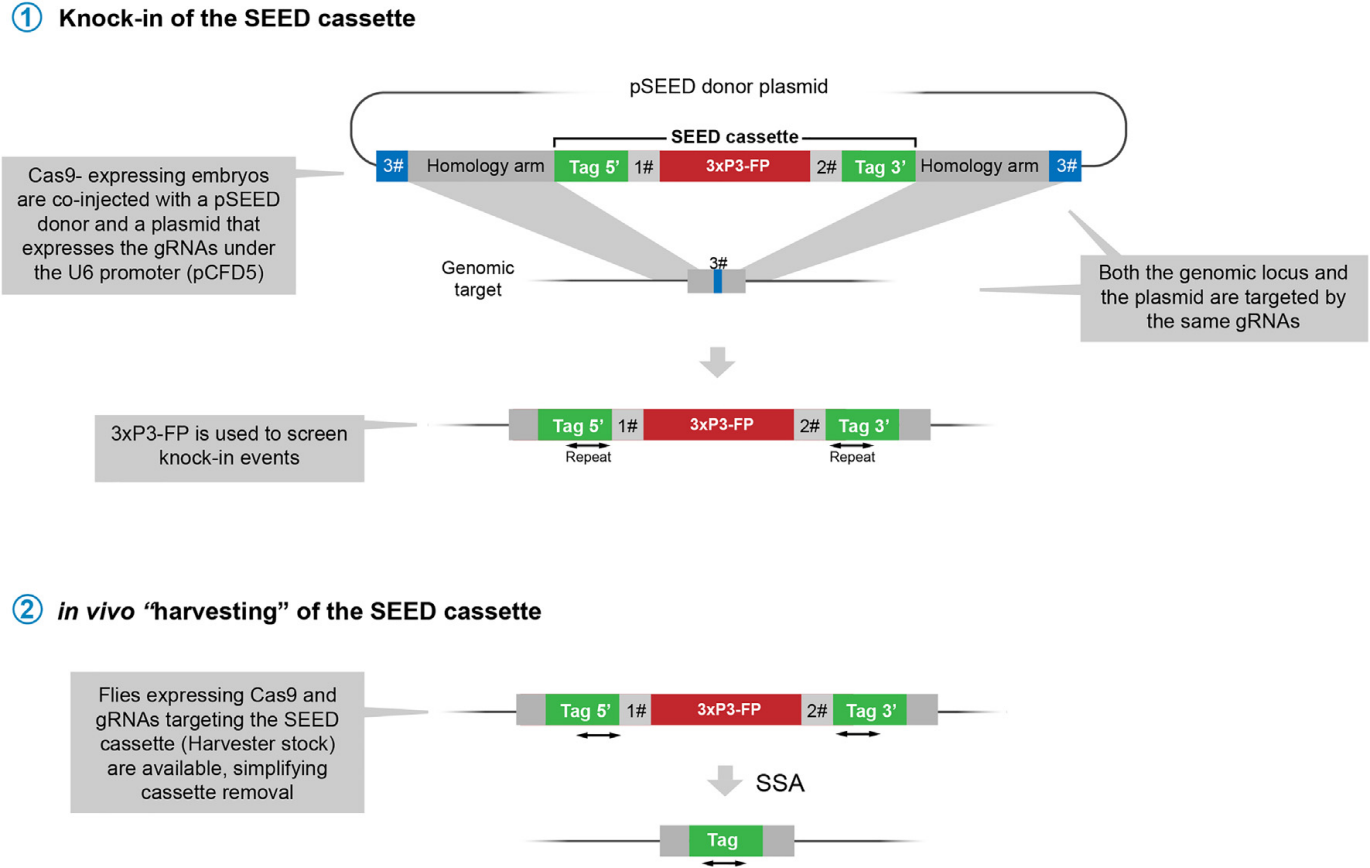
Main steps of SEED/Harvest genome editing

The SEED/Harvest technology depends on two CRISPR events. The first one is responsible for the integration of the SEED cassette containing the "tag" and a selectable marker for screening. This step requires injection of both the repair template and the gRNA-expressing vector (Figure 1.1). The selectable marker of SEED cassettes is either 3xP3-dsRED or 3xP3-GFP. Both markers are strongly expressed in different developmental stages,^1^^,^^2^ as well as in adult flies. Flanking the marker, the tag to be inserted is split in two parts, 5′ and 3′, sharing an identical sequence repeat between 100 and 400 base pairs. In order to induce the seamless rearrangement of the cassette in the second step (Figure 1.2), the target sequence for two gRNAs not present anywhere else in the Drosophila genome is added in-between the marker and the tag (indicated as 1# and 2# in Figure 1). CRISPR-mediated cleavage of these targets induces repair of the SEED cassette via the Single-Strand Annealing pathway (SSA), seamlessly removing the screening marker (see Aguilar et al. for details^1^). To accelerate this step, transgenic flies are available simultaneously expressing Cas9 and both 1# and 2# gRNAs.

Here, we describe the basic steps for a successful design of in-frame knockins. The protocol focuses on the specifics of the SEED/Harvest technology but the recommendations in the following sections are broadly applicable to other organisms and gene editing technologies.

### Choosing the correct tag

Depending on the specific downstream application, researchers may decide to knockin different types of tags. Correct selection of the tag is critical for the project’s success. For many tags, ready-to-use SEED cassettes are available. In other cases, a custom SEED cassette would be required, see section “Generate your own SEED cassette” at the end of the protocol.

In the following lines, we offer a small summary of the advantages and disadvantages of each type of tag.

**Figure 2:**
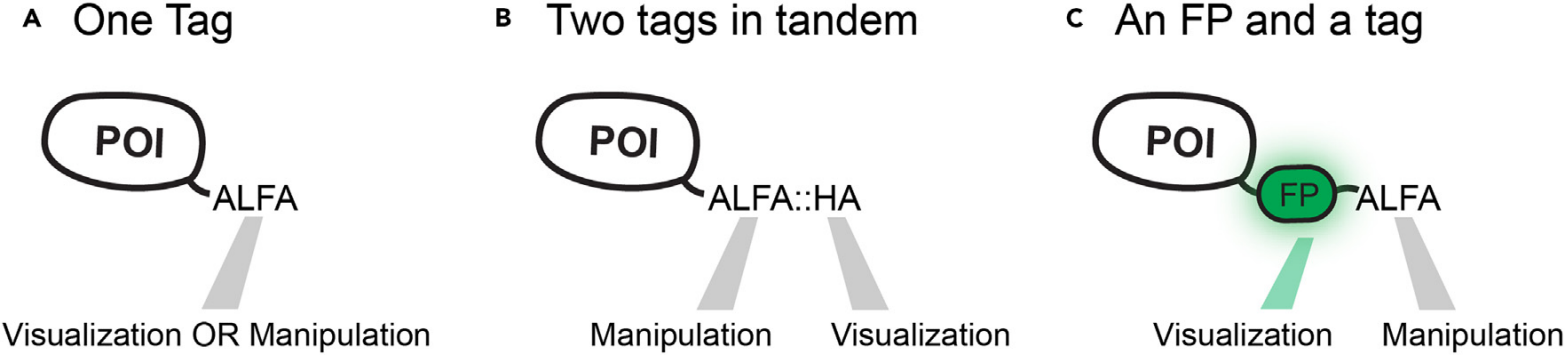
Dual tagging permits simultaneous visualization and manipulation of the target protein

***Note:*** At the time this protocol is written, four SEED cassettes containing FPs are available: sfGFP and mCherry (medium brightness, medium photostability, large amount of protein binders available, widely validated), mGreenLantern (very bright, mid-low photostability, detected by anti-GFP nanobodies) and mScarlet3 (very bright, medium photostability, no protein binders available). In addition, we have generated SEED cassettes containing the fluorescent proteins fused to ALFAtag, providing the means to symultaneously visualize and manipulate the target protein.

***Note:*** The fluorescent properties listed above are based in our own experience, both in live imaging in embryos and fixed tissues at larval stages. These properties might be different in your tissue of interest.

1. Fluorescent proteins (FPs). The development of FPs is a scientific field in constant evolution.^3^ FPs are the first choice when live imaging is intended (but not the only one, see chromobodies in the next section). The main drawback of FPs is their size (often > 20 kDa). While linkers can ameliorate the steric hindrance of FP tagging, for certain targets this may not be optimal (see “Choosing the best tagging position”). In general, we choose the fluorescent protein based on the following criteria:
  a. Optical properties.
    i. *Brightness: In vivo* live imaging requires long-term exposure to high-power lasers. Exceptionally bright fluorescent proteins are preferred to limit laser exposure. Brightness and spectra of FPs can be easily explored in: https://www.fpbase.org/chart/. Nevertheless, brightness determination in purified samples not always correlates with brightness *in vivo.* Thus, we recommend to first investigate which FPs are the brightest in the tissue of interest or to empirically determine it.
    ii. *Photostability:* Hand in hand with laser exposure comes the problem of bleaching. Photostability of fluorescent proteins must be taken into account if long-term imaging or intensity quantification is intended. Bleaching results are hard to extrapolate between different microscopes and the rate of photobleaching and excitation intensity do not linearly correlate; therefore, we advise to take photobleaching measures as a simple indication. Nevertheless, in recent years, this problem is experiencing unprecedented developments.^4^
  b. Chemical properties.
    The addition of any tag can potentially change the chemical properties of the target protein. We recommend the use of monomeric FPs, avoiding tagging with slightly dimeric proteins, such as EGFP, or with tandem-dimeric proteins. In addition, the cellular environment to which the FP is going to be exposed must be taken into consideration (extracellular, nuclear, etc.). In specific cases (tagging lysosomal or endosomal luminal proteins, for example), the sensitivity to pH is a critical factor in the selection of the FP. See Sharma et al.^5^ for a more careful description of FPs properties.
  c. Availability of protein binders.
    Even when the main intended application is live-imaging, we recommend that other possible applications are considered when choosing a tag. Biochemical applications profit from the availability of highly specific antibodies and other protein binders (nanobodies, DARPINs, etc.). In addition, there is a growing wealth of methods to manipulate proteins directly *in vivo* by using protein binders that recognize FPs.^6^^,^^7^ GFP (and close derivatives) currently possess the best availability of protein binders among FPs.^8^^,^^9^ Alternatively, consider using a FP and a small tag in tandem (See Figure 2).

**CRITICAL:** If tissue-specific labeling is intended, only SEED cassettes encoding FPs can be used (See Aguilar et al.^1^). While fluorescent tags result in a truncated fluorescent protein unless rearranged, the shorter peptide tags are always incorporated in both rearranged and non-rearranged cassettes.

2. Short peptide tags.
  First characterized and used for biochemical studies, short tags provide a wide range of uses. The selection of the short tag depends on the intended application. These applications can be broadly grouped into these classes.
  a. Biochemical studies. These include immunoprecipitation or immunoblotting, among others. The existence of high-quality ready-to-use commercial reagents for each application will be decisive.
  b. *In situ* immunodetection. Not all antibodies are suited for their use in immunostaining. We recommend to confirm the suitability of the antibody in the preferred tissue before choosing an epitope/antibody pair. Recently, fluorescent-conjugated nanobodies offer a powerful alternative to conventional antibodies (due to their small size and improved penetrance into tissues). These might be the best option when performing super-resolution studies.^10^ In contrast, conventional antibodies offer more signal amplification.
  c. *In vivo* visualization. Genetically encoded fusions of a FP and a protein binder, also referred as chromobodies, have been proven a valuable approach for the analysis of protein dynamics *in vivo*.^11^ Chromobodies permit cell-specific labeling of the target protein, opening the door for numerous new applications. However, for the success of this approach *in vivo* it is critical to utilize conditionally stable protein binders.^1^
  d. *In vivo* manipulation. The high intracellular stability of nanobodies and other protein binders (engineered scFvs, DARPins, monobodies, etc.) make them ideal candidates for the direct *in vivo* manipulation of proteins. Manipulations include miss-localization, degradation or enzymatic manipulation, among others. For a complete commentary on their use in flies, see Aguilar et al.^12^ and Lepeta et al.^13^

Here we provide a simple table to guide decision making (Table 1).

***Note:*** Currently, three SEED cassettes encoding short tags are available: pSEED-OLLAS:HA, pSEED-ALFA:HA and pSEED-Moontag:HA. In general, we consider ALFA:HA the preferred tag, as it offers a good balance between visualization and manipulation. For extracellular detection, OLLAS:HA was also successfully employed.^14^

***Note:*** We recommend to use always more than one type of tag in tandem for each targeting event. This will permit simultaneous manipulation and visualization of the target protein (Figure 2). In addition, it increases the chances of successful immunostaining (highly variable between tissues and antibodies). So far, we have included HA in all short-peptide cassettes since, in our hands, we find it one of the best epitope tags for tissue-immunostaining.

**Figure 3:**
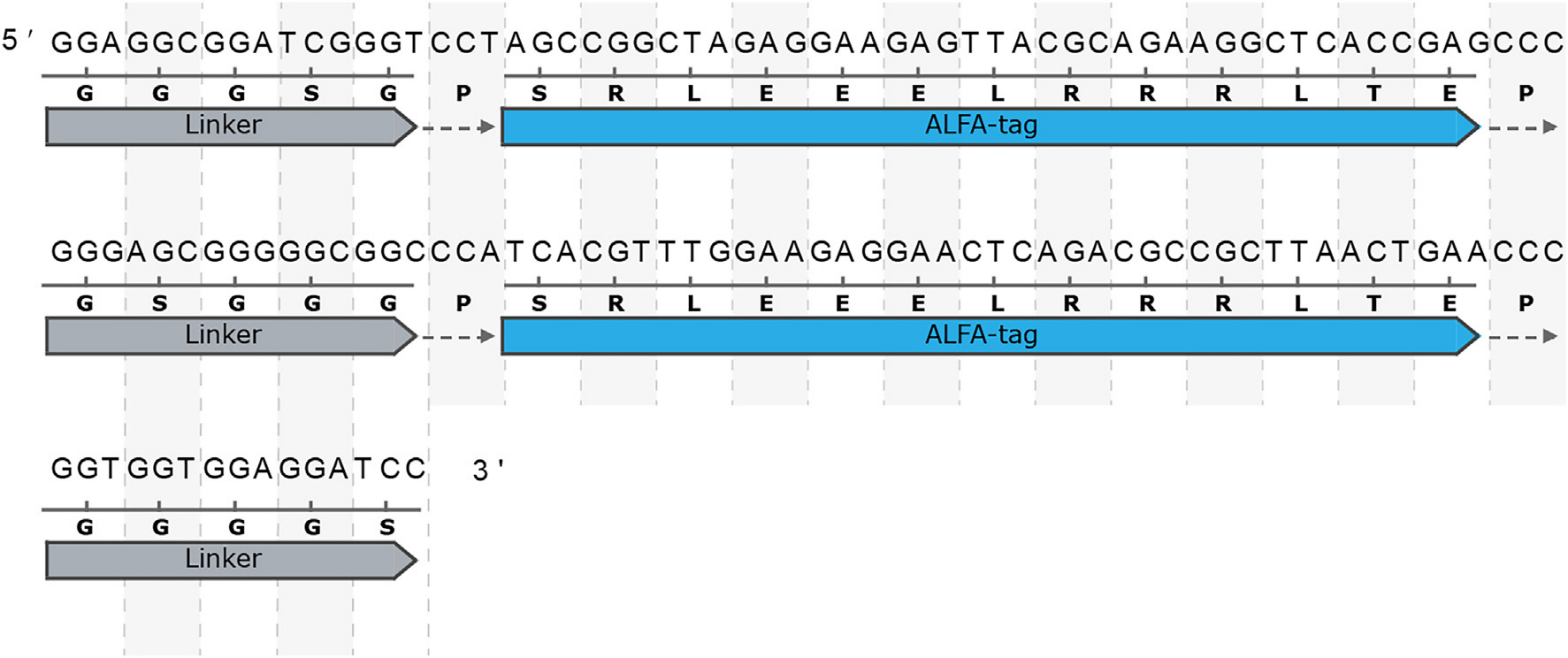
Example of codon variability to avoid problems during SSA

***Note:*** The short size of these tags permits the addition of several units of the same type without a dramatic increase in size. If the protein has very low expression, the inclusion of two or three copies of the same tag might be beneficial. Nevertheless, one must take into consideration several points. 1) If *in vivo* visualization via chromobodies is intended, the recruitment of the chromobodies (each >35 kDa) will result in an increase of the size of the protein. This is especially critical when using long arrays.^15^ 2) Protein manipulation does not require multiple tags.^16^ 3) When performing SEED/Harvest, avoid using many repeated sequences (by choosing alternative codons when possible, see for example Figure 3), to avoid interfering with the SSA-mediated step.

**Table 1:** Short peptide tags available and their properties

Name	Sequence (size)	Protein binder available (for intracellular expression)	Antibody commercially available	Best for	Comments
HA	YPYDVPDYA (9aa)	YES^17^	YES (Several providers)	Tissue immunostaining Biochemical studies	Under certain circumstances, can be cleaved^18^
FLAG	DYKDDDDK (8aa)	YES^19^	YES (Several providers)	Biochemical studies	Highly charged tag (7 charged aa)
ALFA	SRLEEELRRRLTE (13aa)	YES^20^	YES (https://nano-tag.com/shop/)	*In vivo* protein manipulation/visualization Biochemical studies	The tag adopts helical structure.
Suntag	EELLSKNYHLENEVARLKK (19aa)	YES^15^	YES (Several providers, C11L34 clone)	Study of protein synthesis	
Moontag	KNEQELLELDKWASL (15aa)	YES^21^	NO	Study of protein synthesis	
V5	GKPIPNPLLGLDST (14aa)	YES^22^	YES (Both antibodies (several providers) and nanobody (Chromotech) are available)	Tissue immunostaining	
Myc	EQKLISEEDL (10aa)	NO∗	YES (Several providers)	Biochemical studies	Detects cMyc protein ∗While the Nb is available, it has not been shown to work as an intrabody. Not extensively characterized.^23^
OLLAS	SGFANELGPRLMGK (14aa)	NO	YES (Several providers, L2 clone)	Tissue immunostaining	
SPOT-tag	PDRVRAVSHWSS (12aa)	NO∗	YES^10^ (Chromotech)	Tissue immunostaining (Best in bivalent form, see^10^)/ Biochemical studies	Also called BC2-tag, It also binds to endogenous human β-catenin^10^^,^^24^ ∗While the Nb is available, it has not been shown to work as an intrabody
127D01-tag	SFEDFWKGED (10aa)	YES^25^	NO	*In vivo* protein manipulation	While no commercial Nb are available, protocols for Nb production and use have been published.^26^ Cross-reacts with the human CXCR2 protein.
VHH05Tag	QADQEAKELARQIS (14aa)	YES^25^	NO	*In vivo* protein manipulation	While no commercial Nb are available, protocols for Nb production and use have been published.^26^ Cross-reacts with the human UBC6e protein.
PepTag	AVERYLKDQQLLGIW (15aa)	YES^27^	NO	*Immunoprecipitation, live-cell microscopy*	Derived from HIV’s gp41. Previously named VHH2E7. Helical structure
EPEA	EPEA (4aa)	NO∗	YES	*Affinity purification, flow cytometry*	Tag only works when fused to the C-terminus of the protein. Also knows as C-Tag. Also detects α -synuclein. ∗While the Nb is available, it has not been shown to work as an intrabody

3. Other peptide tags.

FPs and short tags are the most widely used moieties for endogenous tagging, but other types are available. Tagging with split fluorescent proteins^28^ has also been proposed in Drosophila.^29^ This strategy permits tissue-specific labeling. However, this technology is limited to protein visualization, and not optimized for biochemical studies or direct, *in vivo* manipulation. Another alternative could be self-labeling protein tags (HaloTag, SNAPTag, etc.). They have been demonstrated to efficiently label proteins *in vivo* in a number of contexts.^30^

4. SEED/Harvest also permits the introduction of other features, such as transcriptional reporters, etc. As an example, we also generated a T2A-Gal4 SEED cassette.

**CRITICAL:** When generating your own SEED cassettes, make sure that no BsmBI sites are included within sequence of the tags. BsmBI sites will be later used for donor assembly.

### Choosing the best tagging position: Analyzing the locus

Genomic architecture is one the first critical factors that must be taken into account when attempting endogenous tagging. In some cases, all isoforms can be tagged, in others only a subset. If different isoforms exist, tagging position needs to be carefully evaluated not only at the protein, but also at the gene levels. In general, C-terminal targeting results in tagging of most isoforms,^31^ but there are multiple examples that do not follow this rule. Here, we provide an example of the decision making of the gene *nervana 2 (nrv2).*

1. In the J-browser (or similar genome browser, UCSD, Ensembl, etc.), identify the number of putative isoforms of your target. For J-browser, this track is denominated “Gene: transcript view”. If only one isoform has been described, the decision of tagging can be taken purely on the properties of the target protein.

**Figure 4:**
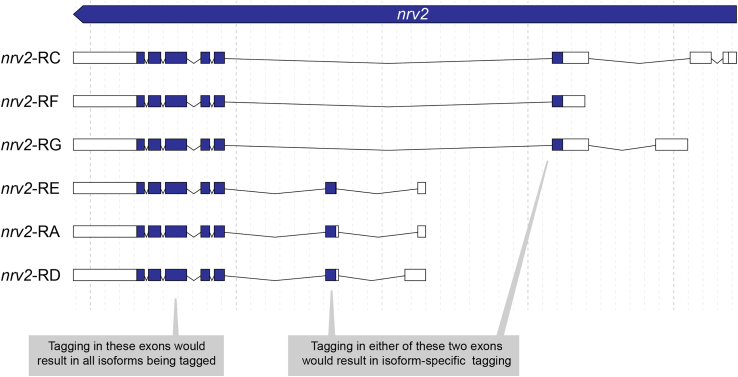
Genomic architecture of *nrv2* Protein coding exons are highlighted in dark blue, UTRs in white.

Example: 6 isoforms have been predicted for nrv2, containing 4 different transcription start sites. All 6 isoforms share the same 3′ exons (See Figure 4).

2. If more than one isoform is present, identify the common protein-coding exons. It is possible that different isoforms only differ in their untranslated regions (UTRs) but not in their protein coding sequence (CDS). Further information on the different isoforms and polypeptides produced (their exact sequence, if they are experimentally validated, etc.) can be obtained from FlyBase in the section “Gene models and products” of your target gene.

Example: in spite of its many isoforms, only two putative nrv2 CDSs are identified. The CDS of RA, RD and RE isoforms is identical and so are the CDS of isoforms RC, RG and RF.

3. Decide whether a subset or all isoforms are to be tagged. This decision will be based on the intended application and the specific biological question that is been interrogated. For many applications tagging of all isoforms is desired. In other cases, different isoforms may be labeled with different tags or only the isoform expressed in specific cells is labeled. If not tagging all isoforms, we recommend analyzing the high-throughput expression data of the target gene. This is available in J-Browse by activating the different “expression” tracks.

Example: nrv2 isoforms express differently across tissues. For example, among the gonads of males and females, the longest isoforms (RC and RG seem to be only expressed in the testis).

### Choosing the best tagging position: Analyzing the protein

1. Tagging position: Traditionally, most proteins are tagged either in the N- or C-terminus. This decision depends mainly on the available literature about the chosen protein. While tagging at these positions is normally the first choice, many proteins present functional domains in either end. In these cases, tagging at the wrong position may result in disruption of protein function. For example, 30% of the tested C-terminally-tagged Flyfos fTRG lines, disrupted protein function.^31^ In addition, proteins tagged in the N- or C-terminus using the same tag often present wide differences of protein localization (and presumably function).^32^^,^^33^ Finally, it is possible that these residues are not exposed on the surface of the folded protein, or that the protein contains one or several protease cleavage sites. In all those cases, the identification of internal tagging sites is of great importance.

***Note:*** A thorough literature analysis can save months of work. Carefully check if there are previously described tagging positions for your target protein and its homologs in different systems.

**CRITICAL:** Secreted proteins often present a signal peptide that is cleaved after protein translocation into the endoplasmic reticulum. When tagging these proteins in the N-terminus, the tag must be positioning after the cleavage residue. The signal peptide can be predicted using: https://services.healthtech.dtu.dk/services/SignalP-6.0/.^34^ Take into account that some signal peptides are not easily predicted.

2. PTM and other protein features. We recommend to first obtain an overview of the different domains and posttranslational modifications (PTMs) of the targeted protein. In general, we find UniProt (https://www.uniprot.org/) an excellent database for a preliminary analysis. Several factors have to be taken into account, such as the localization of predicted domains, confirmed and predicted PTMs, or the topology in the plasma membrane (if any), among others.

**CRITICAL:** The presence of protease cleavage sites may be a critical factor when choosing tagging position. For example, many proteins are produced as pro-proteins and later cleaved, during their biogenesis or in other stages of the protein's life-cycle. We advise a careful literature search, especially for secreted proteins. For the prediction of Furin cleavage sites, we recommend ProP^35^ (https://services.healthtech.dtu.dk/services/ProP-1.0/).

**Figure 5:**
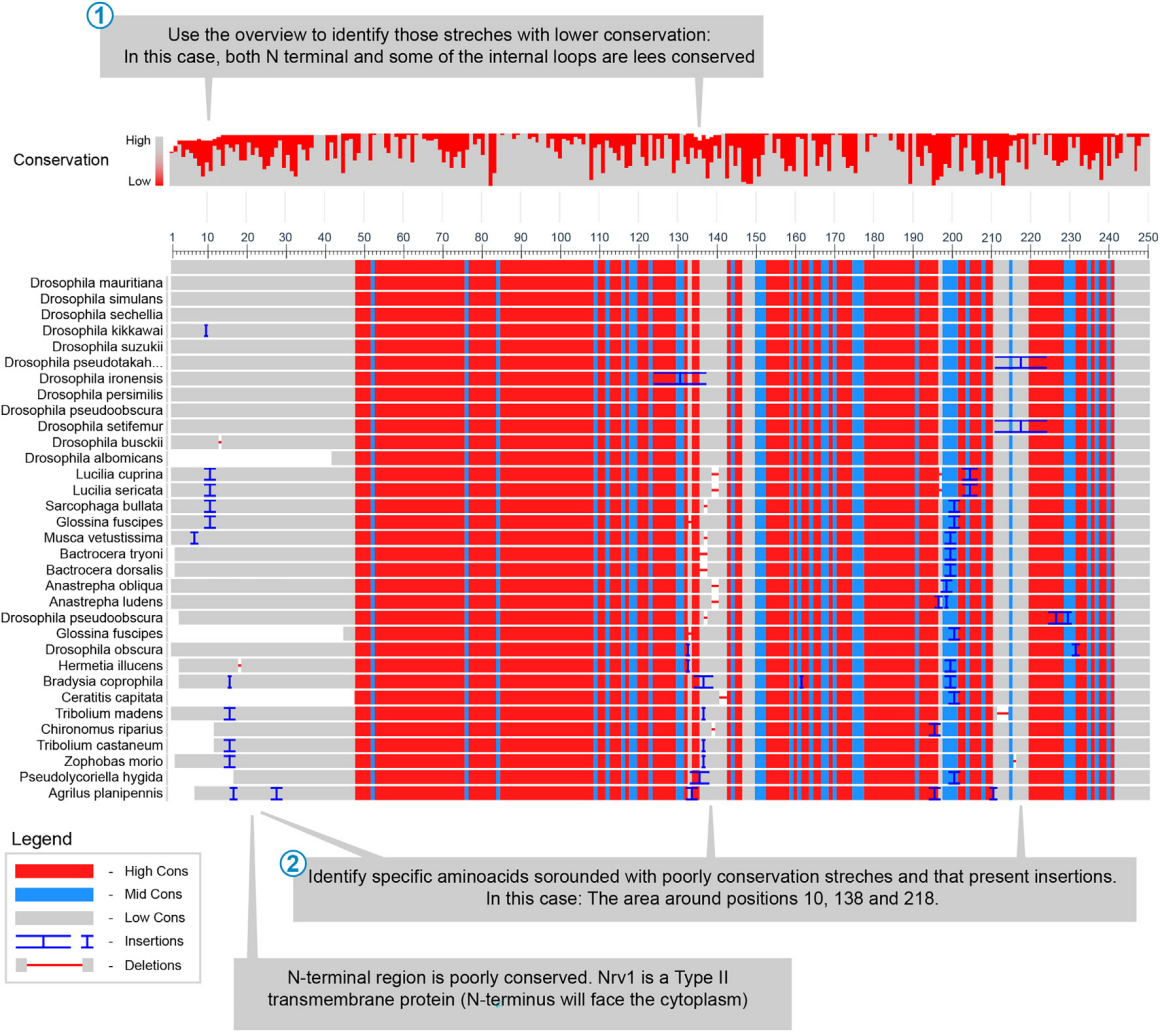
Conservation analysis of Nrv1 protein using the MSA viewer For convenience, only the first 250 amino acids are shown.
  b. Structural analysis.
    i. Confirm that the insertion points are exposed in the protein and do not affect structural features. Insertions affecting structural features of the protein’s core are likely to result in partial or complete loss of function. Thus, it is essential to confirm that those less conserved regions selected by sequence analysis are located within flexible loops. Today, the structures of a large number of proteins have been experimentally determined and are available through the PDB database (https://www.rcsb.org/). For those cases in which the structure of the protein remains unknown, we recommend to validate tagging positions using AlphaFold (https://alphafold.ebi.ac.uk/) or other prediction servers. Structural information of the proteins is also available through FlyBase in the entry of the coding gene, heading: “*gene models and products*”, subheading: “*structures*”.
      Example: Nrv1 positions identified by conservation analysis are placed in areas of the protein that are exposed to the solvent (Figure 6) and are not part of any predicted structural feature. If no additional information is available, and if C or N terminal tagging of this protein would not be possible, we would opt for extracellular tagging after amino acid 137 and after amino acid 10 for intracellular tagging. Nrv1is a subunit of a well described complex. Thus, it would be advised to check if structural analysis of the complex is available. Indeed, such structures exist (PDB:4RET) for one of the Nrv1 orthologs. Preliminary analysis of this structure confirmed that the chosen loop does not participate in complex formation in that context.

3. Internal tagging: In order to identify the best internal tagging positions, we take a combined approach, in which we first evaluate the protein’s primary sequence, identifying the sites with highest variability among related species and then evaluate the effect insertions would have on the secondary structure. Here we provide the tagging of Nrv1 protein as an example.
  a. Sequence-based prediction of insertion-tolerant regions. Conserved proteins accept a limited degree of change while preserving its original function. Mutations in the functional core of a highly conserved protein often result in the partial loss of function. However, most proteins present regions that are less sensitive to change, making the latter ideal to introduce tags. In order to identify these regions, we compare the primary sequence of our target to that of their orthologs in other insect species.
    i. In the Blast website (https://blast.ncbi.nlm.nih.gov/Blast.cgi), select “Protein Blast” and paste the protein sequence of the target protein. Then, under the heading “chose search set”, subheading “organism”, introduce “*Drosophila melanogaster (taxid:7227)*” and mark the box “*exclude*”. Click “*Blast*”.
    ii. Select the Multiple sequence alignment viewer (MSA viewer) and spot those sequences with higher variability. Stretches with one or several insertions in closely related species are often ideal places for tagging.
      Example: Nrv1 contain 309 amino acids (Figure 5). Conservation analysis revealed that the N terminal part is less conserved. Being a type II transmembrane protein, this domain will face the cytoplasm. In other related insects this domain is even missing. Within this N-terminal tail, there seems to be a hotspot of small insertions and deletions around amino acid 10‒15. Within the displayed area, two other regions present less conserved sequence stretches.

**Figure 6:**
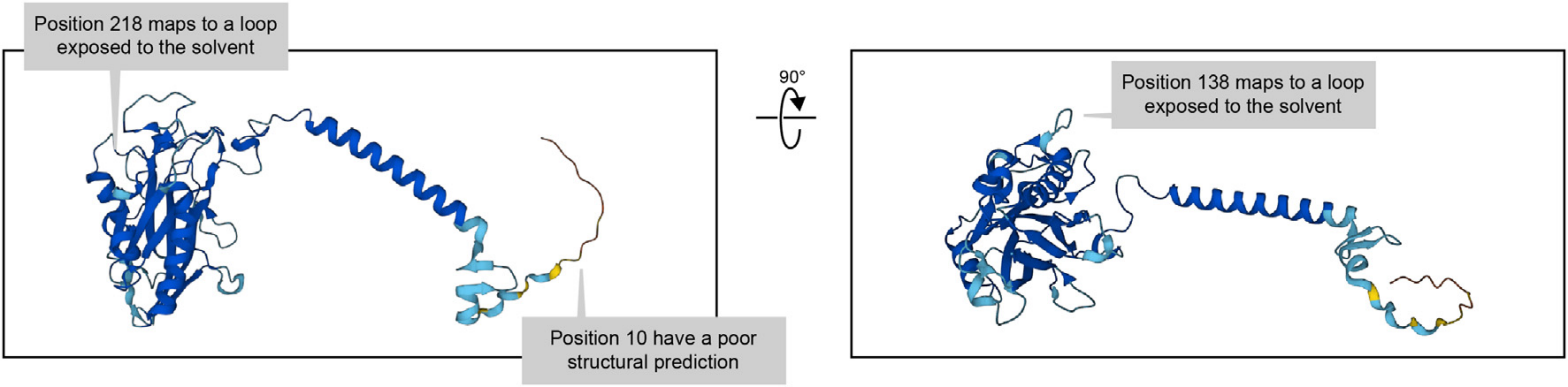
Structural analysis based on AlphaFold
  c. Integrated structure-sequence analysis. An alternative way to display conservation scores on structural protein models is the use of ConSurf^36^ (https://consurf.tau.ac.il/). This bioinformatic tool permits the prediction of functional residues and analysis of conservation scores using solely the UniProtID as an imput. UniProtIDs of Drosophila proteins can be easily retrieved from the FlyBase gene Entries under the heading “g*ene models and products*”, subheading “*Polypeptide data*” in the table “*Annotated polypeptides*”.
    ***Note:*** This approach is best for identifying poorly conserved stretches in the target protein. It does not, to our knowledge, highlight the presence of insertions. We recommend combining this approach with the first one.

### Peptide linkers

Linkers between the tag and the target protein can also ameliorate the steric effect of the tag insertion. Depending on the cloning technology employed, careful linker design has not always been a priority. It is not uncommon to find linkers including restriction sites, attL sites, or even multiple-cloning-site leftovers. All these features can present unexpected properties *in vivo* and must be avoided.

Linkers can be broadly classified into natural and synthetic.^37^ In general, we prefer the use of synthetic linkers, as their properties are easier to predict. In addition, natural linkers might present some protein-protein interacting surface that interferes with the function of the tagged protein. Synthetic linkers can be classified into flexible linkers, ideal to allow the least steric hindrance on the target, and rigid linkers, ideal to prevent the interaction between the target protein and the tag. Most of the insertions that we have generated using SEED/Harvest harbor flexible linkers, as they are generally used to visualize the target. Our preferred linker is the “GlySer” linker, one or several repeats of the GGGGS sequence. Both Gly and Ser are small amino acids, which are thought to provide increased flexibility.^38^ The inclusion of Ser, as opposed to the Gly only linker, is believed to offer increased stability in aqueous environments, as this polar amino acid can form hydrogen bonds with water. For an in-depth review on protein linkers see: Chen et al., 2013.^37^

***Note:*** Most available SEED cassettes include a simple two amino acidic Gly-Ser as linker. This linker must be extended on one or both ends depending on the tag position within the target protein. Even when tagging in the N- or C-terminus, we preserve these amino acids after the Met or before the stop codon.

***Note:*** Separation between tags is also fundamental to allow simultaneous recognition of both tags by their respective antibodies/protein binders. We recommend a separation of at least one GGGGS between tags.

**CRITICAL:** SEED/Harvest is based on the SSA repair pathway, which depends on the sequence homology around the DSBs. To increase the desired outcome, linker sequence must be as divergent as possible at both sides, in order to avoid undesired SSA outcomes. Take this into account when designing SEED cassettes (see Figure 3).

### Co-insertion of a tag and other features

In some occasions, the intended knockin might not only include the insertion of a tag, but also the introduction of one or several amino acid changes.

**Figure 7:**
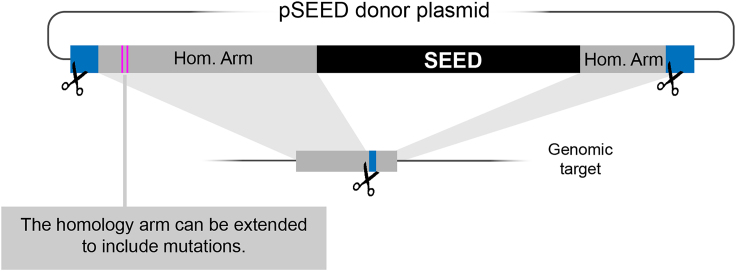
Mutagenic homology arms

If the intended change is in the vicinity (<3 kb) of the tagging position, changes can be easily introduced in the homology arms (Figure 7). In principle, longer homology arms are also possible, but in our hands, cloning larger homology arms than 3 kb is often problematic.

If the tagging position is far away (>3 kb) from the intended mutagenesis, we recommend a two-step knockin strategy. First generate one of the knockins following this protocol until the establishment of harvested knockins. Subsequently, combine the generated insertion with *nos*-Cas9 and proceed with the knockin of the second feature. If many different manipulations are intended in a single CDS, and the gene of interest has a complex exon structure, we recommend to use one of the established protocols to insert a “landing site” and introducing the variants by φ C31-mediated transgenesis.^39^

**Figure 8:**
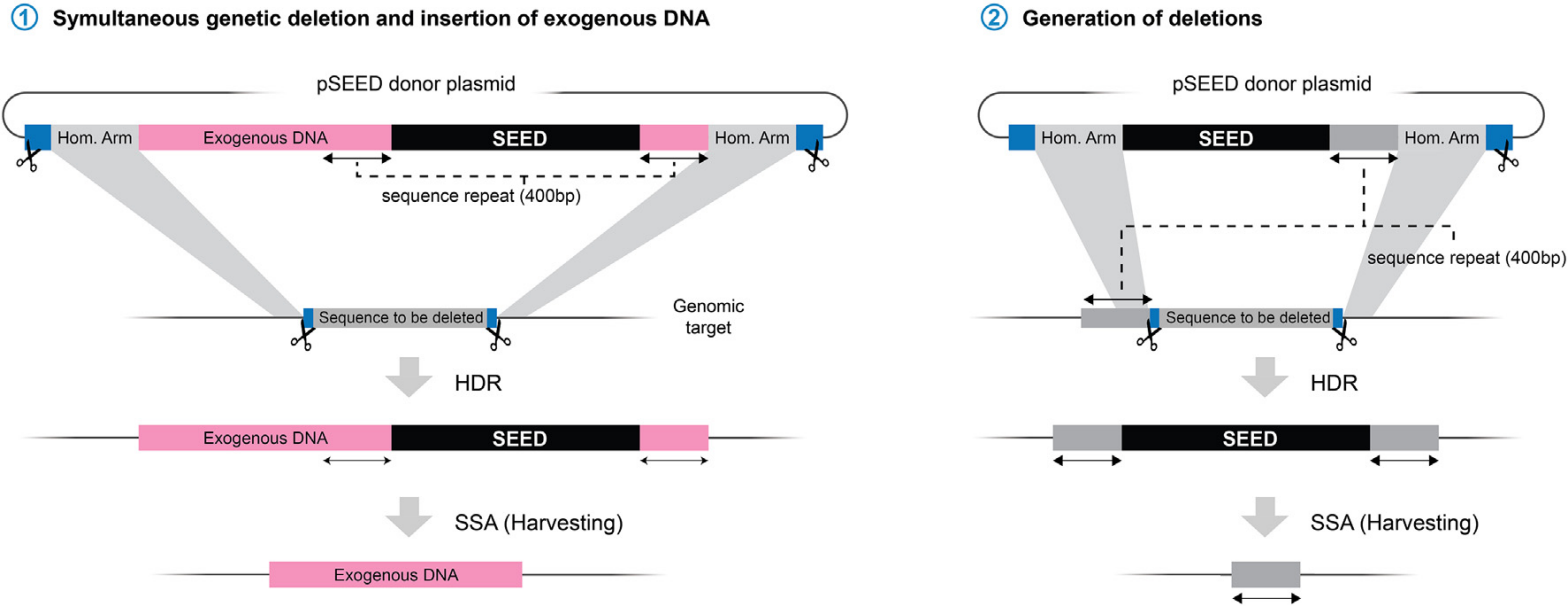
Possible usage of SEED to delete and insert sequences simultaneously or make genomic deletions Note that it is possible that the second variation proposed could, in principle, generate the several outcomes by HDR. One of these outcomes being the generation of the deletion in one step without the integration of the SEED cassette, the other the intended SEED insertion. These multiple outcomes might reduce the efficiency of integration.

***Note:*** SEED/Harvest can be easily adapted to insert many different features, not only protein tags. Among the possible applications is the substitution of a part or whole locus by another sequence. In such cases, it is essential that the homology arms anneal in areas flanking the deletion to be introduced. The exogenous sequence to be inserted can then be introduced at both sides of the cassette as mention in the introduction. We recommend keeping the repeats for SSA in the larger end (around 400 bp). Some examples of possible modifications of SEED are presented in Figure 8.

### Preparation and analysis of target sequences

In order to effectively design and confirm knockins, we recommend first creating a digital map of the targeted area using your preferred DNA analysis platform (SnapGene, Benchling, SerialCloner, etc.). These maps should normally include the exonic and intronic information and specific features such as splicing acceptors or starting and STOP codons.

Raw or decorated FASTA files can be downloaded from FlyBase “Sequence Downloader” tool (http://flybase.org/download/sequence/). Simply introduce the *flybaseID* of your gene (FBgn), select *type*: “*sequence features*”, and download “*all FASTA”*. These files can be opened with any DNA analysis platform. Alternatively, the sequence downloader can be accessed from the FlyBase entry of each gene, under the heading “*genomic location*”, sub-heading “*sequence*”, by clicking “*get sequence*”. Ensembl genome browser (www.ensembl.org/) also offers an intuitive sequence download tool, under the tab “*export data*”. ºThis map will be used to design the gRNAs, align the sequencing results, etc. The UCSC genome browser (https://genome.ucsc.edu) is also very well suited for retrieving genomic information.

**CRITICAL:** Since gRNA recognition may be impaired by mutations in the specific stock, we highly recommend to sequence the targeted area in the selected fly strain (e.g. nos-Cas9), especially if there is no whole genome sequence data for that strain. The use of sequenced strains is also preferred to identify possible off-targets.

***Note:*** to minimize possible confusions, we recommend setting the sequence viewer in such a way that the gene of interest is in the top strand in the direction of transcription; for genes in the bottom strand, this will require “flipping” the sequence from its original direction.

## Key resources table

**Table:** 

REAGENT or RESOURCE	SOURCE	IDENTIFIER
**Bacterial and virus strains**		

TOP10 electrocompetent *E. coli*	Thermo Fisher Scientific	C404050

**Chemicals, peptides, and recombinant proteins**		

Q5 polymerase	NEB	M0491
OneTaq Hot Start Quick-Load 2X master mix	NEB	M0488
BbsI	NEB	R0539
Gibson assembly master mix	NEB	E2611
NEBridge Golden Gate Assembly (BsaI-HF v2)	NEB	E1601S
NEBridge Golden Gate Assembly (BsmBI-v2)	NEB	E1602S
dNTP mix 10 mM	Sigma-Aldrich	D7295
DNA gel loading dye 6x	NEB	B7021
1 kb DNA ladder	Solis BioDyne	07-12-0000S
Nuclease-free water	Ambion	AM9937
GelRed nucleic acid gel stain	Biotium	41003
Proteinase K	Macherey-Nagel	740506
Agarose	Merck	9012-36-6
T4 DNA ligase reaction buffer	NEB	B0202S

**Experimental models: Organisms/strains**		

*y*^[1]^*v*^[1]^ ; P{y[+t7.7] v[+t1.8] = nos-Cas9.R}attP2	BDSC	78782
*y*^[1]^*v*^[1]^ ; P{y[+t7.7] v[+t1.8] = nos-Cas9.R}attP40	BDSC	78781
*w* ; Dr^[1]^/TM3, Sb^[1]^ Ser^[1]^ P{w[+mC]=attR.nos-Cas9, U6:3-1+2sgRNA.attR}FS18 (Harvester)	BDSC	Bloomington: 604565
*y*, *w*	N/A	N/A

**Oligonucleotides**		

*U6_fwd*, 5′-ATT TTC AAT TTA ACG TCG GGG-3′		
*T3_fwd* 5′-GCA ATT AAC CCT CAC TAA AGG-3′		

**Recombinant DNA**		

pSEED-sfGFP	Aguilar et al.^1^	Addgene: 219639
pSEED-mCherry	Aguilar et al.^1^	Addgene: 219657
pSEED-mScarlet3	Aguilar et al.^1^	Addgene: 219640
pSEED-mGreenLantern	Aguilar et al.^1^	Addgene: 219643
pSEED-ALFA:HA	Aguilar et al.^1^	Addgene: 219646
pSEED-OLLAS:HA	Aguilar et al.^1^	Addgene: 219647
pSEED-MoontagHA	Aguilar et al.^1^	Addgene: 219648
pSEED-HA:EYFP	Aguilar et al.^1^	Addgene: 219656
pSEED (BsaI)	Aguilar et al.^1^	Addgene: 219658
pSEED-mGreenLantern:ALFA	Aguilar et al.^1^	Addgene: 219644
pSEED-mScarlet3:ALFA	Aguilar et al.^1^	Addgene: 219641
pSEED-sfGFP:ALFA	Aguilar et al.^1^	Addgene: 219642
pCFD5	Port et al.^40^	Addgene: 73914

**Software and algorithms**		

SnapGene	Dotmatics	Version 7.0

**Other**		

Gel and PCR Clean-up Kit	Macherey-Nagel	740609
Miniprep Plasmid Purification Kit	Macherey-Nagel	740588
Midiprep Plasmid Purification Endotoxin Free Kit	Macherey-Nagel	740422
Tesafilm	Tesa	04104-00267-00
Cellulose membrane for dialysis, Millipore, type VSWP, 0.025 μm	Merck	VSWP02500
Microloader tips	Eppendorf	5242 956.003
Capillary tubes, borosilicate glass capillaries	Harvard Apparatus	EC1 40-0038
Microscope slides	Thermo Scientific	12342108
Oil for embryo injection	Voltalef, Atofina	H10S
Thermal cycler		
Benchtop centrifuge with thermal control (for 50 mL Falcon tubes =		
Microcentrifuge with thermal control (for 1.5 and 2 mL)		
25°C incubator		
Stereoscope with fluorescent illumination		
Electrophoresis chamber and power source		
Trans-illuminator for DNA gels		
NanoDrop spectrophotometer		
Collection chambers	Custom made	
Needle puller	Sutter Instrument	Model P-97
Microinjector pump	Eppendorf	FemtoJet
Inverted microscope	Leica Microsystems	DMIL
Mechanical micromanipulator		
Binocular	Leica	M60
Leica Leitz microscope mechanical micromanipulator	Leica Microsystems	Leitz

## Materials and equipment

**Table:** Fly squishing buffer

Reagent	Final concentration
Tris-HCl pH 8.2	10 mM
NaCl	25 mM
EDTA	1 mM
Proteinase K	200 μg/mL
ddH_2_O	-
Total	

Premixed stock of squishing buffer without proteinase K can be prepared beforehand and stored at RT. Store proteinase K at ‒20°C and add before squishing

• TAE buffer: 40 mM Tris, 20 mM acetic acid, 1 mM EDTA. It can be stored at room temperature for years.
• LB media: For one liter: 10 *g* Tryptone, Yeast Extract 5 *g*, Sodium chloride 10 *g*, MilliQ Water. Autoclave before use. It can be store at room temperature for years.
• Embryo glue: fill a 50 mL falcon tube with tesafilm tape. Fill the gaps with hepatane and rotate at room temperature 12–16 h. The next day, distribute the liquid phase in 1.5 mL plastic tubes and centrifuge 10 min at 14.000 rpm. White precipitate pellets at the bottom. Only apply supernatant to the slides. It can be stored at ‒20°C for years.
• Grape juice plates for embryo collection: For 80 plates (1 L): in 750 mL of MilliQ water, mix 21.2 *g* of Agar (Bacteriology grade any brand) and 12.45 *g* of Sucrose (Food-grade). Heat to 90°C and autoclave for 20 min at 121°C. In parallel, mix 2 *g* of Nipagin (Methyl 4-hydroxybenzoate) and 250 mL of Grape juice and stir up under heat until Nipagin is totally dissolved. Add the water, agar and sucrose and mix until it becomes homogeneous. Let it cool down to 56°C and aliquot 12 mL in 60 mm plates. Solidify the plates in a stack at room temperature for up to 16 h. Plates can be stored at 4°C for up to a month.

## Step-by-step method details

### gRNA design

**Timing 30 min**

**Figure 9:**
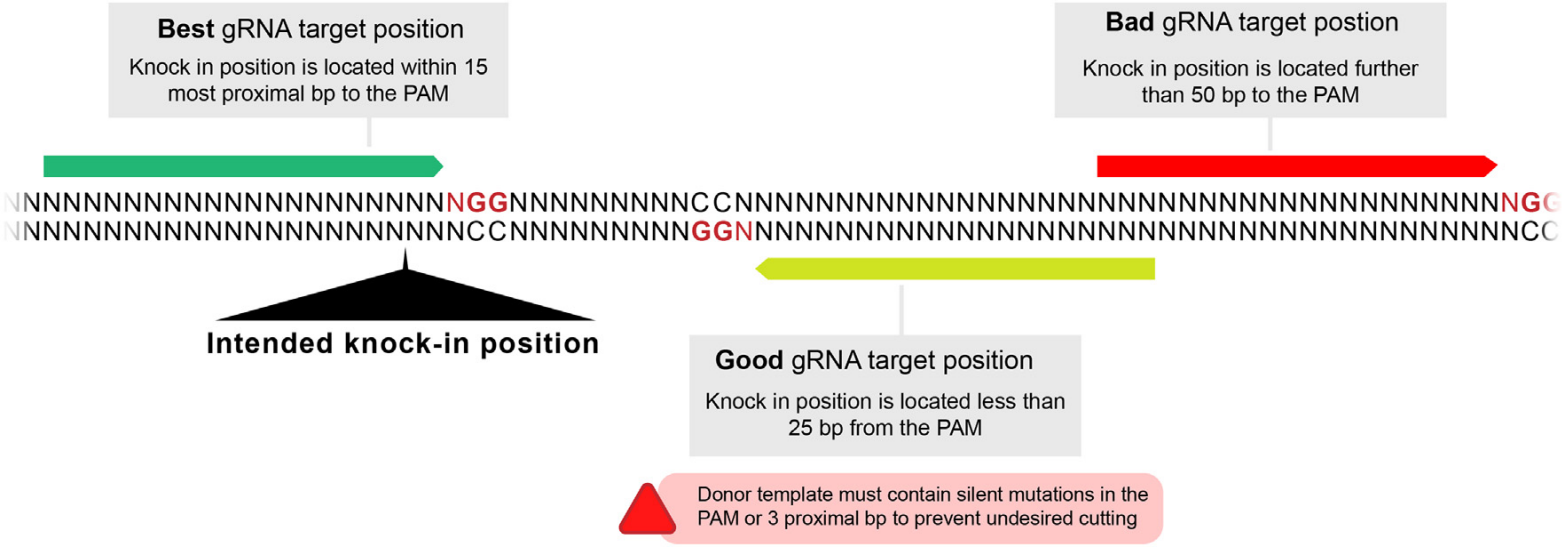
gRNA design principles

***Note:*** It is possible that the target region does not contain gRNA candidates following this approach. In those cases, set “Low stringency” in the search properties of the Target finder website.

***Note:*** It is possible that no suitable Cas9 targets are available in the target region. In such cases, consider using Cas12a enzyme. Suitable Cas12 variants have recently been optimized for their use in Drosophila.^46^ Cas12a relies on a T-rich PAM sequence.

1. Identify sgRNA target sites using the CRISPR Optimal Target finder (http://targetfinder.flycrispr.neuro.brown.edu/).^41^ Select the genome of the chosen flies (in this protocol, nos-Cas9 ChIII) and supply the sequence of the targeted region. Set the guide length in 20 bp, high stringency, and “NGG” only PAM (protospacer adjacent motif). www.flyrnai.org/crispr3/ ^42^ also provides a robust alternative for evaluating the presence of gRNA target sites in given sequences. The latter option also provides other sequenced Cas9 fly genomes.
2. Select one or two gRNAs with no off-targets and high efficiency scores (in that order of priority). If off-targets are unavoidable, select gRNAs with off-targets located on a chromosome different from the one bearing the target gene. Ideally, the chosen gRNA(s) target sequence is disrupted by the knockin integration in the proximal region of the PAM (Figure 9).^43^ In cases in which the knockin insertion does not disrupt the gRNA target, the sequence of the donor plasmid must be mutated to prevent undesired cutting (See “*Donor design”*). When possible, we avoid targeting sites located further away than 50 bp from the insertion point.^44^^,^^45^

### gRNA cloning

**Timing: 2 days**

This section describes how to generate the gRNA-expressing plasmids.

**Figure 10:**
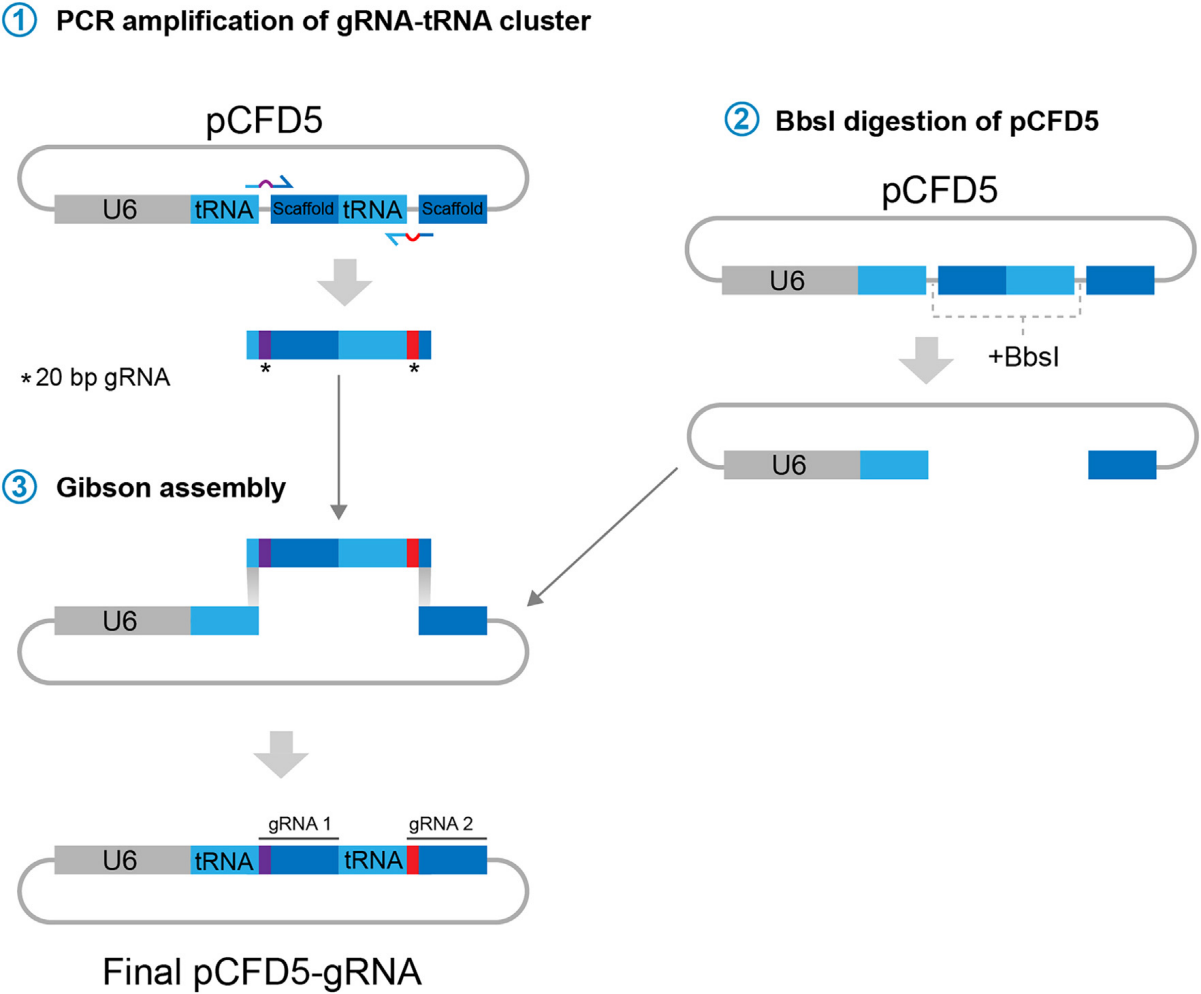
gRNA cloning workflow

***Note:*** We routinely use two gRNAs per knockin. We do this to increase our chances of success in case one of them does not cut. However, similar success has been observed using only one. Both gRNAs are designed based on Figure 10 and do not need to be positioned in specific ways respect the knockin position. We adopted this section from Port and Bullock 2016.^40^ The original version also contains the cloning protocol for one and more than two gRNAs. Protocols are available here: http://www.crisprflydesign.org/wp-content/uploads/2016/07/pCFD5cloningprotocol.pdf and in.^40^ Briefly:

3. Design and order the following primers (Figure 11):

**Figure 11:**
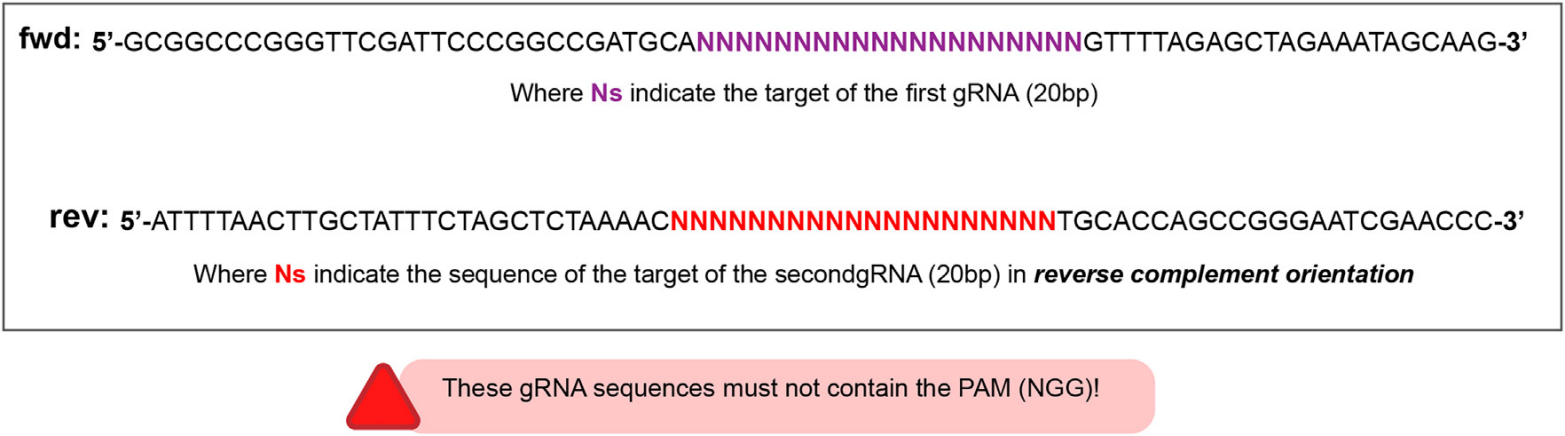
Primer design
4. Set up the following PCR reaction:

**Table:** 

Reagent	Amount (μL)	Final concentration
5X Q5 buffer	5	1X
Forward primer (10 μM, see Figure 11)	1	0.4 μM
Reverse primer (10 μM, see Figure 11)	1	0.4 μM
dNTP mix 10 μM	1	400 μM
pCFD5 3 ng/μL	0.5	60 pg/μL
Q5 DNA polymerase	0.5	0.04 U/μL
Nuclease-free water	16	
Total	25
  ***Note:*** It is very important to use a good proofreading polymerase. We recommend the use of Q5 polymerase

**Table:** 

Number of cycles	Denature	Anneal	Extend
1	98°C, 30 s		
24	98°C, 10 s	61°C (+0.5°C/cycle), 10 s	72°C, 15 s
6	98°C, 10 s	72°C, 10 s	72°C, 15 s
7			72°C, 2 min

  a. Run the digested product in a 1.5% agarose gel following standard procedures. Correct size for the fragments is 234 bp.
  b. Cut and purify the band using the Gel purification Kit following manufacturer’s instructions. Elute in 25 μL Nuclease free water.
5. Digest at least 5 μg the pCFD5 vector with Bbs1 in NEBuffer 2.1 at 37°C for > 4 h using the following information:

**Table:** 

Reagent	Amount (μL)	Final concentration
10× NEBuffer 2.1	5	1x
pCFD5 vector (1 μg/μL)	5	500 ng/μL
Bbs1 (10 U/μL)	1.5	0.3 U/μL
Nuclease-Free water	38.5	
Total	50

  a. Run the digested product in a 1.5% agarose gel following standard procedures. Correct linearized size of the plasmid is 6.6 Kb.
  b. Cut and purify the band using the Gel purification Kit following manufacturer’s instructions. Elute in 20 μL Nuclease free water. Expected concentration is around 50–100 ng/μL.
    ***Note:*** keep the digested pCFD5 at ‒20°C for future gRNA clonings.
6. Set up the following Gibson assembly reaction in ice:

**Table:** 

Reagent	Amount (μL)	Final concentration
2× Gibson Assembly master mix	6	1x
BbsI-digested pCFD5 vector (from step 5) 50 ng/μL	1	4.2 ng/μL
gRNA fragment 1 (from step 4) 3.5 ng/μL (∼3 M excess respect vector)	1	290 pg/μL
Nuclease-Free water	4	
Total	12

  a. Incubate at 50°C for 1 h, immediately place on ice.

### Bacterial transformation

**Timing: 2 h**

***Note:*** It is important to use high efficiency competent *E*. *coli* bacteria (>10^7^ cfu/ug). We use home-made electrocompetent Top10, but other electro- or chemical-competent bacteria can be used.

7. Dialyze 5 μL of the product for 15 min on a cellulose membrane placed on water. If performing chemical transformation, this step is not necessary.
8. Electroporate the resulting product into electrocompetent bacteria following manufacturer’s instructions.
  **CRITICAL:** For optimal quality of transformation, we recommend the addition of media (normally 700 μL of LB) immediately after electroporation.
  **CRITICAL:** When using a freshly BbsI-digested aliquot of pCFD5 vector, transform 0.25 μL of digested backbone (50 ng/μL) as a control of digestion.
  a. Recover the cells at 37°C while shaking at 600 rpm in a thermoblock for 1 h.
  b. Plate the transformed cells on LB/agar plates supplemented with 100 μg/mL ampicillin. Incubate 12–16 h at 37°C.

### Plasmid preparation and sequencing

**Timing: 3 days**

9. Next day, pick two or four colonies and set up 3 mL LB cultures supplemented with 100 μg/mL ampicillin. Incubate 12–16 h with shaking at 37°C. Do not discard the plates as they might be needed for troubleshooting later on.
10. Purify plasmid from the cultures using the Miniprep kit following manufacturer’s instructions. Use 2 mL for purification and store the rest at 4°C.
11. Sequence the Miniprep via your Sanger sequencing provider using the primers *U6_fwd* or *T3_fwd*.
  a. If the sequence is correct, gRNA recognition sequences (20 bp) will be integrated before each of the gRNA scaffolds, use the remaining liquid culture stored at 4°C in step 10 to set up 110 mL LB cultures supplemented with 100 μg/mL ampicillin.
  b. Incubate 12–16 h with shaking (180 rpm) at 37°C.
12. NOTE: As an alternative to direct sequencing, a pre-screening can be performed by colony-PCR using primers primers *U6_fwd* and *T3_fwd.* The expected outcome is a band of 985 bp. For a full protocol on this process check: http://www.crisprflydesign.org/wp-content/uploads/2016/07/pCFD5cloningprotocol.pdf..
13. Purify the plasmid out of 100 mL of culture with a Midiprep kit following manufacturer’s instructions. Elute with 200 μL–350 μL of endotoxin free water. Measure the concentration using a (NanoDrop) spectrophotometer. The plasmid is now ready to be prepared for injection.

***Note:*** Other laboratories inject routinely miniprep-quality DNA with success. Nevertheless, we have not extensively compared both qualities using SEED/Harvest. It is possible that the high insertion rates of SEED/Harvest remain unaffected by this change.

**Pause point:** Plasmids can be stored at ‒20°C for years. Optionally, glycerol stocks can be made for long-term storage (‒70°C) and convenient re-culture of the bacteria.

### Donor generation

**Timing: around 8 days (synthesis), 2 hours (cloning)**

This section describes how to generate the donor plasmids containing the SEED cassettes.

This protocol is intended for having the homology arms synthetized in a plasmid vector compatible with the Golden Gate predesigned SEED plasmids. The available reagents are displayed in the table below.

**Table:** 

Reagent name	Repository number
Plasmids	
pSEED (BsaI)	Addgene: 219658
pSEED-OLLAS:HA	Addgene: 219647
pSEED-ALFA:HA	Addgene: 219646
pSEED-Moon:HA	Addgene: 219648
pSEED-mScarlet3 (3xP3-GFP)	Addgene: 219640
pSEED-mCherry (3xP3-GFP)	Addgene: 219657
pSEED-sfGFP	Addgene: 219639
pSEED-mGreenLantern	Addgene: 219643
pSEED-mScarlet3:ALFA (3xP3-GFP)	Addgene: 219641
pSEED-sfGFP:ALFA	Addgene: 219642
pSEED-mGreenLantern:ALFA	Addgene: 219644
pSEED-EYFP:HA	Addgene: 219656

**Fly stocks**	

*w*: *Dr/TM3, Sb, Ser, nos-Cas9, U6:3-1+2sgRNA* (Harvester)	Bloomington: 604565
*UAS-gRNA1+2* (86Fb)	Bloomington: 604566
*UAS-gRNA1+2* (36B)	Bloomington: 604567

***Note:*** Up to this point, all the recommendations given are applicable to most CRISPR knockins in Drosophila and other animals. From now on the protocol will focus only on the SEED/Harvest technology.

14. Design the custom donor vector based on Figure 12.
  a. Donor vector contains the element to be inserted (in this case one of the SEED cassettes) flanked by homology arms. These homology arms start from the place where the insertion is intended and NOT from the cutting site.
    **CRITICAL:** To avoid cleavage of the donor and/or re-cleavage of the modified allele, make sure the gRNA cannot target the homology arms. If the gRNA target site is disrupted by the insertion of the cassette, no further changes are needed. In cases in which the knockin insertion does not disrupt the gRNA target, introduce silent mutations that prevent gRNA recognition. The easiest way is to substitute one of the Guanines of the PAM. Make sure all these mutations are silent and do not modify the amino acid sequence of the target protein. If changing the PAM is not possible, include one or several mutations in the 12 nt adjacent to the PAM, the so called “seed sequence”.
  b. In between the homology arms, place the indicated BsmBI cassette to later insert the SEED cassettes by Golden Gate assembly.
    ***Note:*** Consider that available SEED cassettes include a 1x(Gly-Ser) linker at both sides. If longer linkers are preferred, they must be included in frame flanking either side of the BsmBI cassette.
  c. In order to trigger *in vivo* linearization after injection, place gRNA target sequences flanking the homology arms. These targets must be the original one used for triggering DSB in the target locus. We routinely use the gRNA target sequence with the highest cutting score of the pair (determined by: https://www.flyrnai.org/evaluateCrispr/). Note that these target sites *include* the PAM sequence.
    **CRITICAL:** To make Golden Gate assembly possible, the homology arms must not contain internal BsmBI recognition sequences. If this is the case, remove these sites by including silent mutations or shorten the homology arms to exclude them. Homology arms can be shortened to up to 30 bp.^1^

**Figure 12:**
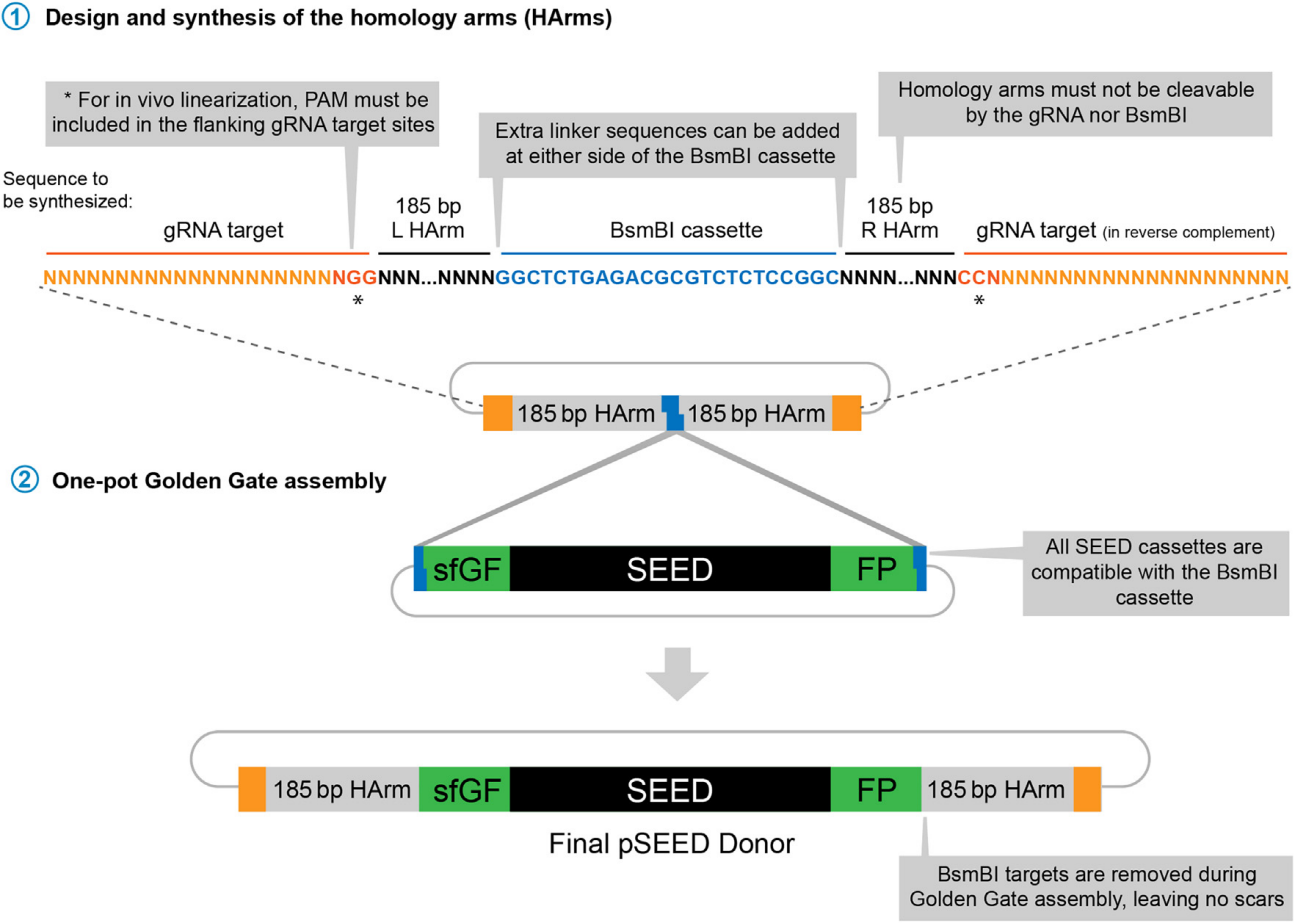
pSEED Donor cloning
15. Order the synthesis of the custom "gRNA target - Homology Arm-BsmB1cassette-Homology arm-gRNA target" sequence from a commercial provider. This sequence should be pre-cloned into a simple vector.

**CRITICAL:** Make sure that your chosen synthesis service clones the fragment in a plasmid that does not contain BsmBI sites. In addition, to ensure easy cloning and DNA preparation, the vector of choice must be small and of high copy number type. We routinely use pUC-GW-Kan or pTwist-Amp-HighCopy.

***Note:*** Some labs may order dsDNA linear fragments and clone them in a small vector. The small price difference with cloned versions might be important when scaling up the method. However, if cloning and sequencing reagents are taken into account, we consider cloned genes a better alternative.

16. In a PCR tube, set up the following Golden Gate assembly reaction:

**Table:** 

Reagent	Amount (μL)	Final concentration
Synthesized plasmid with homology arms (75 ng/μL)	0.5	37.5 pg/ μL
pSEED of choice (75 ng/μL)	0.5	37.5 pg/ μL
T4 DNA ligase Buffer (10X)	1	1X
NEB Golden Gate Enzyme mix (BsmBI-v2)	0.5	-
Nuclease-Free water	7.5	-
Total	10

  a. Incubate in a thermocycler using the following program:
    (42°C, 2 min -> 16°C, 2 min) × 45 cycles then 60°C, 5 min.
17. Proceed with bacterial transformation and plasmid preparation as specified in the points 7 to 12. Sequence the final plasmid using primers in the synthesized plasmid backbone.

**Figure 13:**
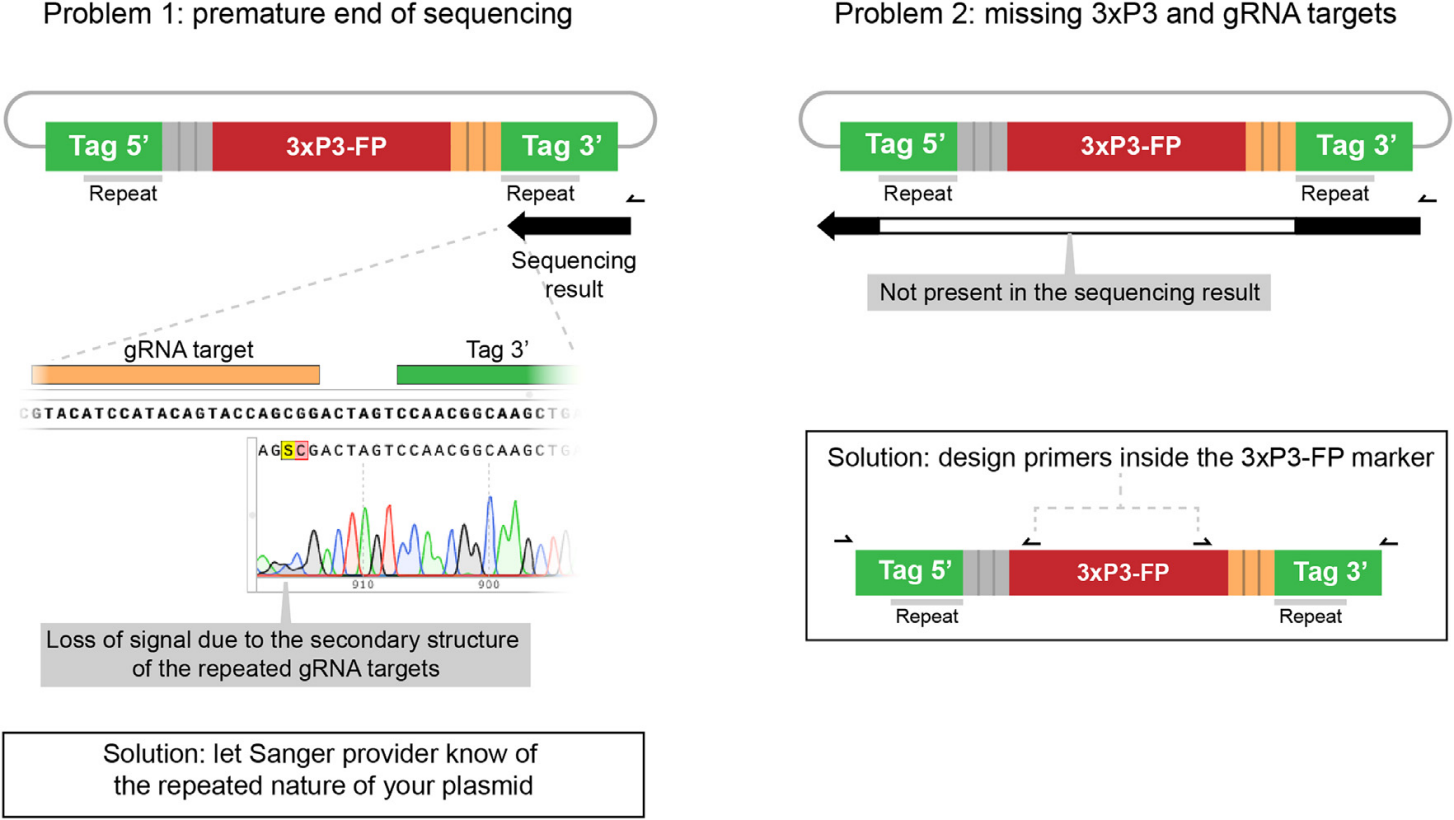
Possible problems when sequencing SEED cassettes

***Note:*** Confirmation of SEED cassettes by Sanger sequencing can sometimes be problematic due to the following problems: 1) Polymerase is incapable to overcome the secondary structure formed by the multiple gRNA target repeats in the SEED cassette (Figure 13, problem 1). This is a common problem that can be overcome by adjusting the conditions of the sequencing reaction. Contact your sequencing provider and specify the presence of repeats in the target sequence. 2) The repeats result in the jumping of the polymerase between the repeats of the cassette (Figure 13. Problem 2). This is far less common, but can arise associated to the previous one. To solve it, internal primers from the 3xP3 marker can be used to sequence outwards from the cassette.

In either case, Golden Gate assembly is based on restriction ligation; therefore, the risk of mutagenesis inside the cassette is minimal.

### Injection

**Timing: 1 h**

In this section, we describe in detail how we inject the pSEED donors and gRNA-expressing plasmids.

Many laboratories are currently outsourcing this step. This option is especially attractive when generating one or few transgenic lines at a time. We find injecting by ourselves a faster and more cost-effective alternative. If you are outsourcing it, make sure sample preparation and concentrations are identical to those described below.

18. Preparing *nos*-Cas9 flies for injections.
  a. In order to ensure that female flies lay well on the day of injection, transfer 200‒400 flies 3 days prior into collection cages placed on grape (or other fruit) juice plates,
  b. keep the flies at 25°C. Supplement the grape juice with a drop of yeast.
  c. For efficient egg laying, change collection cages as well as grape juice plates once a day.
  d. The day of the injection, change the plates 3‒4 times during the morning.

***Note:*** Repeated changing of plates stimulates egg laying and the laying of fertilized eggs that might be retained from the night before, ensuring high embryo quality for injection.

**CRITICAL:** Flies reach their peak of egg laying between their 4th and 14th day. Do not use flies older than two weeks.

***Note:*** the *nos-Ca9* insertion should be in a different chromosome to that of the target locus.

***Note:*** If juice plates are prepared for immediate use, the yeast drop must be dried to avoid flies from getting stuck to it. Fan heaters are ideal for this purpose.

**Figure 14:**
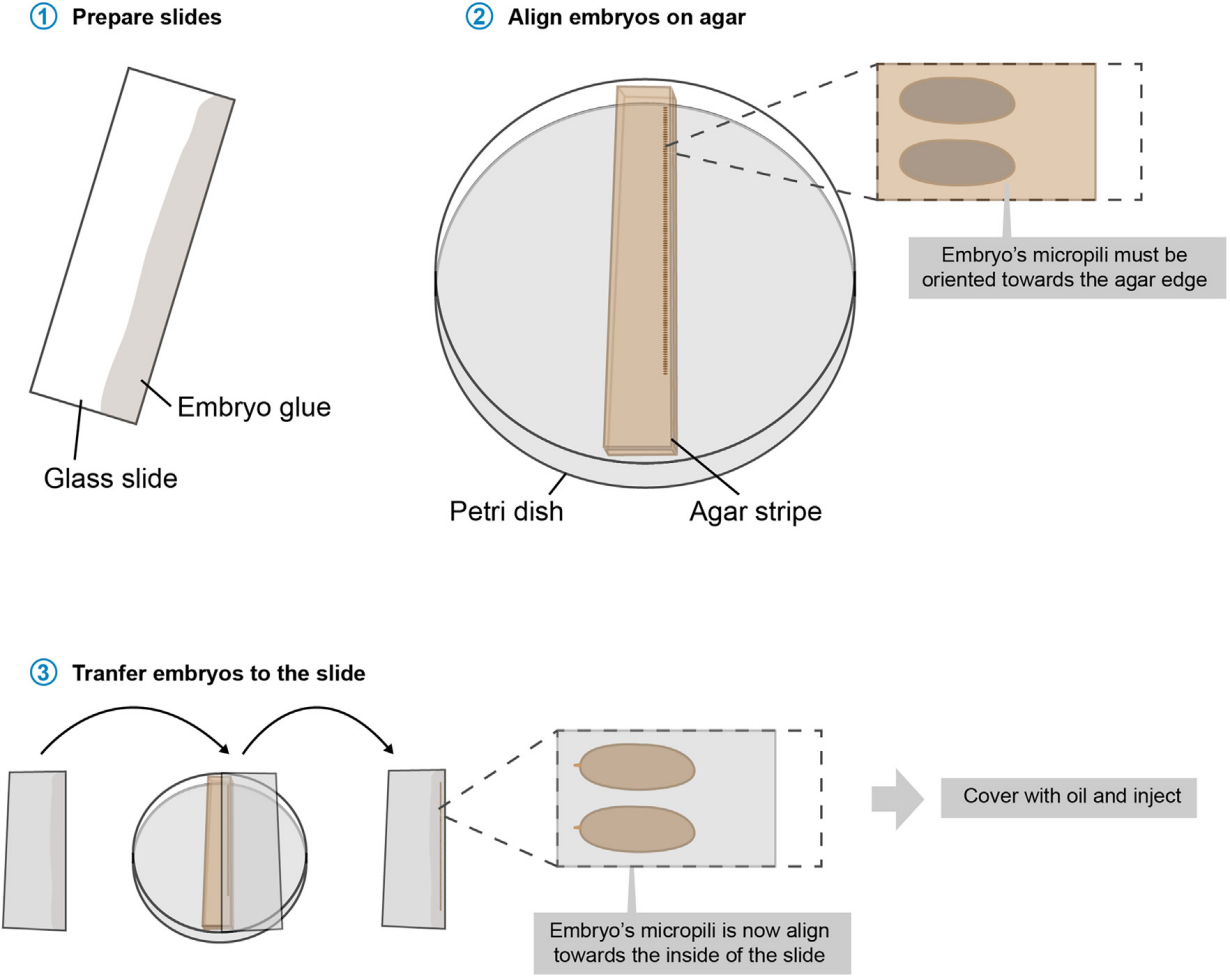
Embryo aligning
22. Embryo alignment and injection:
  There are different injection protocols for embryo injection; this one relies on dechorionated embryos.
  a. Collect embryos for 25–30 min on fresh grape juice plates (+ yeast drop).
    ***Note:*** In our experience, the flies lay best in the afternoon.
  b. Dechorionate the embryos in 3.5% sodium hypochlorite solution for 2 min. After the dechorionation, transfer the embryos with the help of a funnel into a small collection container equipped with a fine-mashed net and wash them thoroughly with water.
  c. Place the embryos on a stripe of LB/agar (without antibiotics) and align them side by side under the binocular. Orient the anterior end (marked by the micropyle) toward the edge of the LB/agar stripe (Figure 14).
    ***Note:*** Before placing the embryos, remove the extra moisture of the agar with a paper tissue to avoid problems in the next step.
  d. Transfer the embryos to the glue-covered glass slide by placing it on top of the agar. Posterior end must now face the edge of the slide. Dry the embryos with a hair dryer (without heat turned on) placed around 20 cm from the slide for 5 min and then cover them with oil to prevent further desiccation.
    **CRITICAL:** When transferring, apply minimal pressure to avoid smashing of the embryos.
  e. Place the slide with the embryos on the microscope. Briefly puncture the embryo by moving the microscope stage to the needle. Small clearing is visible when liquid is injected. Maintain the pump in continuous flow mode and adjust the pressure depending on the break size. To start, we utilize 500 hPa. If the needle is partially clogged, try to fix it by pressing “clear”.
    ***Note:*** After pulling, the needle is closed at its end. When injecting the first embryo, it will break slightly in its tip. This is not perceptible by eye, but will permit the liquid to start flowing out.
  f. Keep the injected embryos in a humid chamber at 18°C for ∼24 h. We place slides inside a sealed chamber with wet paper in which the slide is slightly tilted, with the embryos in the lower end. Upon hatching, this slight ramp will promote most of the larvae to crawl up on the slide, remaining on the same side of the slide and facilitating collection later.
23. Next day, in the late afternoon, place the humid chamber at 25°C 12–16 h.

***Note:*** Alternatively, the embryos can stay in the humid chamber for 72 h at 18° (convenient for Friday injections). Temperatures may vary in different laboratories and might need to be empirically tested.

19. Needle pulling.
  Needles are prepared following manufacturer’s instructions on a horizontal needle puller.
  a. The position and shape of the heating filament as well as the batch of capillaries will condition the final settings. As a reference, our pulling conditions are (in a P-97 puller from Sutter instruments): P = 500, Pull = 150, Vel = 70, Time = 250.
  b. The ramp value, the melting temperature of the capillaries, is determined via a ramp test following manufacturer’s instructions.
    ***Note:*** Needle quality is critical for successful injection. When setting up the puller for first time (or after exchanging the heating filament) we recommend to first determine the ramp value and subsequently generating several needles varying the temperature in three digits at a time. Later, empirically test the different needles until selecting the most appropriate. A complete guide to needle design can be downloaded here: https://www.sutter.com/PDFs/cookbook.pdf
    ***Note:*** Needles can be prepared and stored for months in proper cages. To avoid the contact of the tip with any surface, we store them in closed cages with a central support of plasticine.
20. Sample preparation.
  a. In a 1.5 mL plastic tube, dilute the donor plasmid and gRNA-expressing plasmid in 1x PBS prepared with EF water at a concentration of 100 ng/μL each in a total volume of 30 μL.
  b. Centrifuge your samples at 15.000 rpm for 30 min at 4°C to avoid precipitates clogging your needle. Use microloader tips to load 3 μL directly into the tip of the needle.
    ***Note:*** When loading your sample, try to avoid air bubbles in the needle, careful tapping with the finger is sufficient to remove them.
  c. Turn on the microinjector and place the needle into the holder at the microscope. Align the needle to the center of the focal plane.
21. Prepare slides for injection. Cover unlabeled 76 × 26 mm microscope glass slides with embryo glue and let them dry for at least 2 min. Apply the embryo glue to only one fourth of the surface of the slide along its long edge (Figure 14).

24. Early next morning, collect the hatched larvae and transfer them onto a 50 × 25 glass coverslip.
  a. Place the slide on a holder that minimizes its surface of contact, to avoid the squeezing of larvae that might have crawled to the opposite side of the slide.
    ***Note:*** We used a glass rod, bended t a U-shape under the Bunsen burner. This holder offers two thin “rails” on which we place the slide.
  b. Under the microscope, collect the larvae one by one using a fine metal needle, depositing them in the coverslip, continently placed nearby on the same holder.
  c. Place the coverslip in a common polenta fly food vial at 25°C.
    ***Note:*** We stir the fly food with a bit of water before placing the coverslip, to facilitate the feeding of the larvae.
  d. Grow them until they reach adulthood around 8–9 days after injection.

### Screening and isolation of knockins

**Timing: 1.5 months**

**Figure 15:**
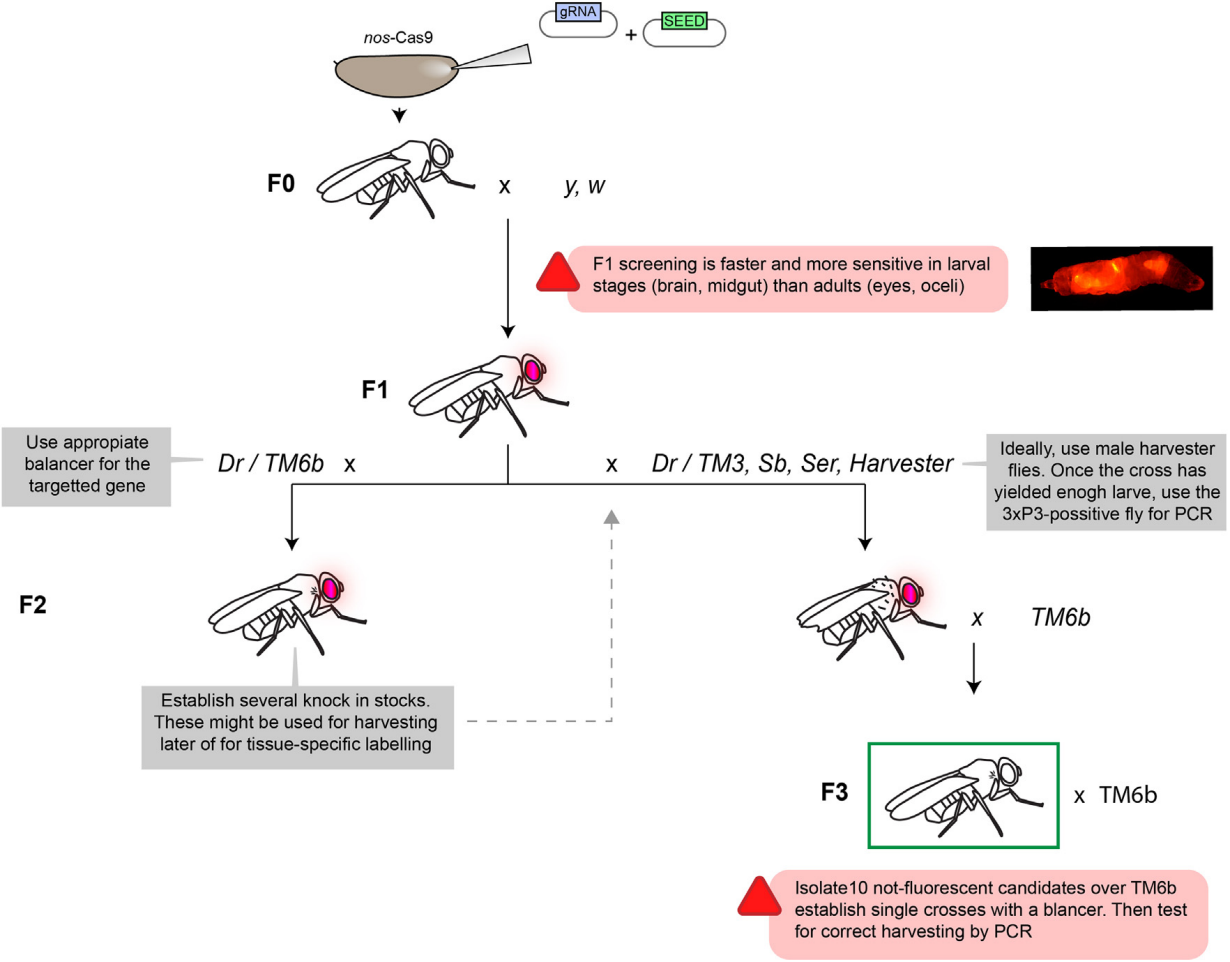
Screening and harvesting of SEED stocks

This section describes how fly stocks containing the correct knockin are isolated (Figure 15).

25. During the days prior to the hatching of the injection survivors, collect enough *y,w* (*yellow* and *white* mutant) virgins and males in time to avoid delays.
  a. Set up single crosses between the survivors and *y,w* flies.

***Note:*** Other strains might be used depending on the desired genetic background of the final stock. We keep most of our flies in this genetic background.

26. Successful integration of the SEED cassette can be detected in the F1 generation by screening larvae or adults for the presence of either 3xP3-dsRED or 3xP3-GFP (dependent on the SEED used).

***Note:*** Both markers are strongly expressed in the eye (and its precursors), brain and midgut, at different developmental stages as well as in the adult. If, as we propose, screening is done during larval stages, place some fluorescent candidates in fresh tubes.

***Note:*** 3XP3-driven fluorescence intensity will greately vary for different loci. Even for the same locus, it is possible that different lines give rise to different intensities. For the same injection, wide divergence between the 3xP3 pattern of different individuals is often an indicator of off-target integration. The same way, if many or all lines display the same intensity and pattern of expression, they are often genetically identical.

**CRITICAL:** It is often easier to detect 3xP3-dsRED in larvae.

27. Cross 10 fluorescent candidates to balancer flies in individual crosses. Depending on the knockin location, different balancer flies must be used. The segregation of the knockin allele can be easily followed using fluorescence.

**Pause point:** Established SEED stocks can be kept at 18°C without removal of the SEED cassette.

***Note:*** Alternatively, set up the crosses directly between the 3xP3-positive flies and the Harvester stock (Step 35). In this case, to be able to follow the knockin in the next generation, it is important that harvester flies are males (to avoid removal of the cassette by maternally provided Cas9).

### Confirmation of precise insertion by PCR

**Timing: 2 h**

**Figure 16:**
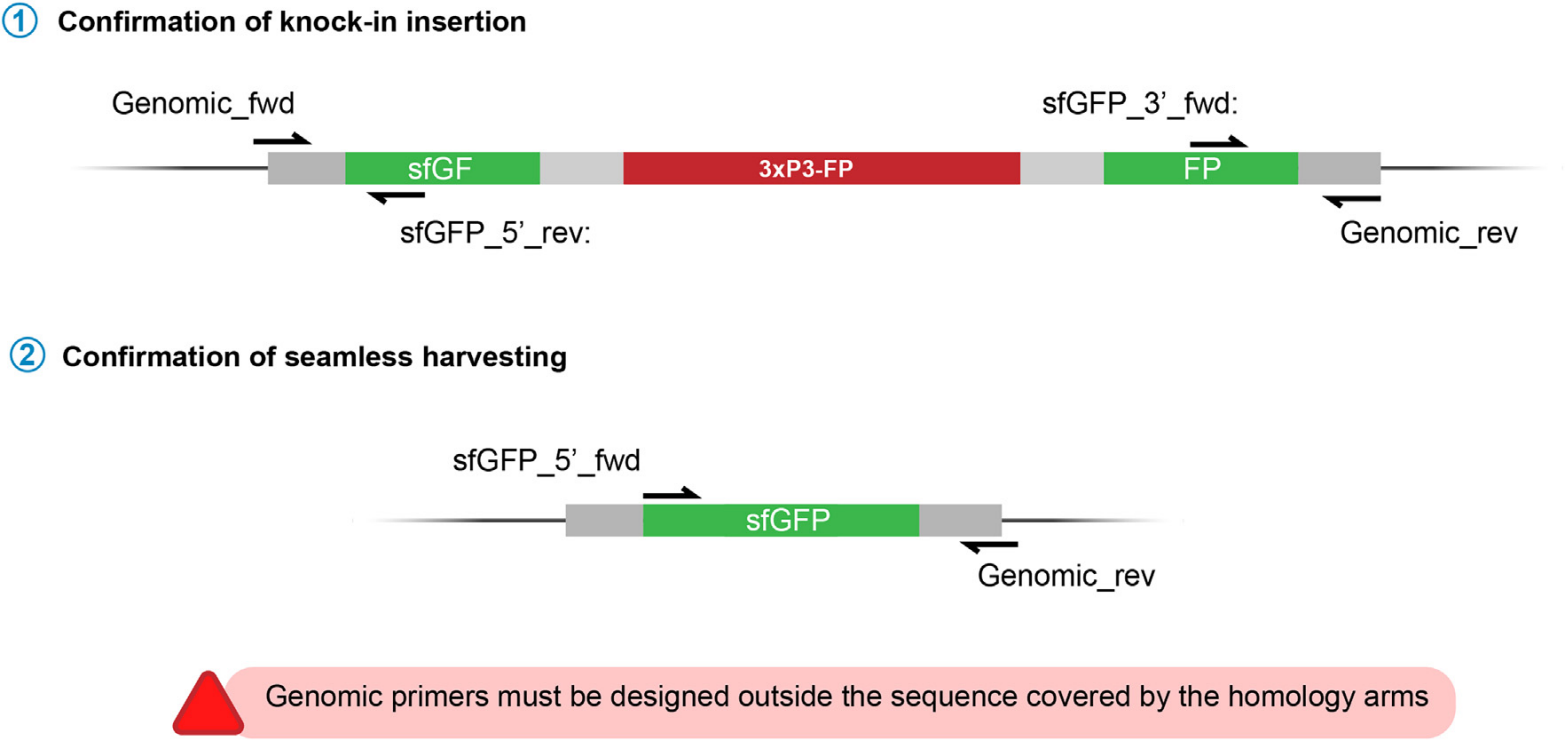
Confirmation of insertion by PCR 1. Two independent PCRs are set up to confirm the 5′ and 3′ ends of the initial knockin. In this case, the sfGFP insertion is used as an example. Genomic primers must be designed ad hoc for the locus of interest, sfGFP primers are available in the primer list, as are those used to sequence the other tags. 2. Primers used to sequence the final insertion after harvesting of the marker. More than one band might be present; select those with only one band in the gel. Final insertion can also be checked with genomic primers, but this strategy might result in favoring the smallest product, the un-tagged copy of the gene, when testing heterozygotes.

28. Generally, we test the fluorescent fly after the cross with the balancer, or from the progeny resulting from each crossing of step 26 using PCR.
29. Preparation of fly extracts.
  a. Transfer single anesthetized flies (or pupae) into PCR tubes. Keep them immobilized by keeping the tubes in ice. Flies can be stored at ‒20°C prior or after DNA extraction.
  b. Add 50 μL of squishing buffer supplemented with proteinase K and crush each of them with a pipette tip or a mini pestle. Try to disintegrate flies very thoroughly to improve DNA extraction.
  c. Incubate the sample for 30 min at 37°C, followed by 2 min at 95°C to heat-inactivate the Proteinase K.
30. Single-Fly-PCR. To cover the 3′ and 5′ ends of the knockin, two different PCRs are set up (see Figure 16). Depending on your choice of tag (e.g., sfGFP), different internal primers are employed (Figure 16). Reverse and Forward primers annealing around the insertion point have to be designed for each gene of interest. For optimal amplification, design primers to result in a putative product of ∼400 bp.

**CRITICAL:** in order to avoid false positive insertions, Genomic_fwd and Genomic_rev primers must be placed outside the sequence covered by the homology arms.

***Note:*** It might be convenient to check for tandem insertions using primers in the insert facing outwards (e.g. sfGFP_5’_rev and sfGFP_3’_fwd in the example of Figure 16).

31. Set up the following reaction on ice:

**Table:** PCR reaction master mix

Reagent	Amount
10 μM Primer F (check Figure 16)	0.5 μL
10 μM Primer R (check Figure 16)	0.5 μL
Template DNA	1 μL
OneTaq 2X Master Mix	12.5 μL
Nuclease-free water	10.5 μL
Total	25 μL

32. Gently mix reaction and collect the reaction at the bottom of the PCR tube by a quick spin.
33. Perform the PCR reactions using these conditions:

**Table:** PCR cycling conditions:

Steps	Temperature	Time	Cycles
Initial Denaturation	94°C	2 min	1
Denaturation	94°C	30 s	24 cycles
Annealing	X (see note) °C	30 s	
Extension	68°C	30 s	
Final extension	68°C	5 min	1
Hold	4°C	Forever

***Note:*** The annealing temperature must be adjusted depending on the melting temperature of the designed primers and that of the selected SEED cassette. Given the wide range of variability of recommended annealing temperatures for the different polymerases, we use https://tmcalculator.neb.com/ for the determination of the annealing temperature.

***Note:*** Other cheap polymerases might be used following manufacturer recommendations. For amplification from single-fly preps, we favor the use of hot-start polymerases pre-mixed with gel-loading buffer.

34. Run 5 μL of the PCR product on a TAE/agarose gel to analyze the product(s).
35. Of at least two positive candidates, run and purify the remaining PCR product (the correct size band) using the Gel purification kit following manufacturer’s instructions. Elute in 15 μL Nuclease-free water and sequence the PCR product via your Sanger sequencing provider using the primers employed for the PCR. It is important to sequence the homology arms boundaries.

**CRITICAL:** Whenever possible, establish at least two independent knockins from different F0 animals. CRISPR editing can lead to unpredictable outcomes and isolating different knockins reduces the risk of identical second off target effects.

***Note:*** Other methodologies have reported considerable levels of integration of the donor backbone in the genome. For SEED/Harvest these events do not seem to represent a major problem, probably due to the sites for in vivo linearization of the donor.

***Note:*** Sequencing through inserted SEED cassettes is subject to the same artifacts as the SEED plasmids (See NOTE in point 16). Notice that PCR amplification using Q5 polymerase does not normally produce these artifacts.

### Harvest the SEED cassette in the germline

**Timing: 15 days**

This section describes how to seamlessly remove the selection marker (harvesting), to obtain a fly stock with the desired final insertion. Outline in Figure 4.

36. In order to remove the SEED cassette and to resolve the seamless gene knockin, cross the confirmed transformants (or directly the isolated 3xP3-positive flies from point 26) with the Harvester Stock (TM3, Sb, Ser, *nos-Cas9, U6-gRNA1#+2#).*
37. Select fluorescent females carrying the insertion over a balancer and the harvester stock. Cross them with a balancer of your choice for the chromosome of your knockin. Male harvester flies are preferred to avoid maternal contribution of the Cas9 and cutting during early embryonic stages.
38. Isolate 10 flies that lack fluorescence and the chosen balancer and cross them with balancers. Once the cross yielded enough progeny, we normally isolate the parents for step 38. Proceed with stock establishment for each line. Avoid selection of flies bearing the harvester chromosome (marked with TM3, *Sb, Ser*).

***Note:*** When harvesting SEED cassettes with shorter repeats (as small protein tags in tandem), It might be beneficial to establish up to 20 crosses. This is because the harvesting rate is reduced respect long repeats and might also be different for each locus. Recently, a new protocol has been proposed to screen positive flies using a piece of their leg.^47^ This can be a good alternative to screen harvested flies before establishing the final stock, reducing fly work.

**Pause point:** Established stocks after removal of the SEED cassette can be kept at 18°C.

### PCR confirmation of the seamless knockin

**Timing: 2 h**

39. After successfully removing the SEED cassette and establishing the fly stocks, another PCR is needed to confirm the seamless genome modification (Figure 16.2).
  a. Prepare Single-fly preps as described in step 29.
  b. Use the Forward and Reverse primers designed in step 30 to start a PCR identical to that of step 30, only varying the primers and using an extension time of 90 s.
  c. Run an agarose gel as previously described and send at least two candidates showing the expected size for Sanger sequencing.

**CRITICAL:** More than one band might be present. If genomic primers are used to test heterozygotes, this is normal. When using one internal primer (as recommended in Figure 16), select those candidates with only the expected band pattern.

### Generation of your own SEED cassettes

**Timing: 15 days**

**Figure 17:**
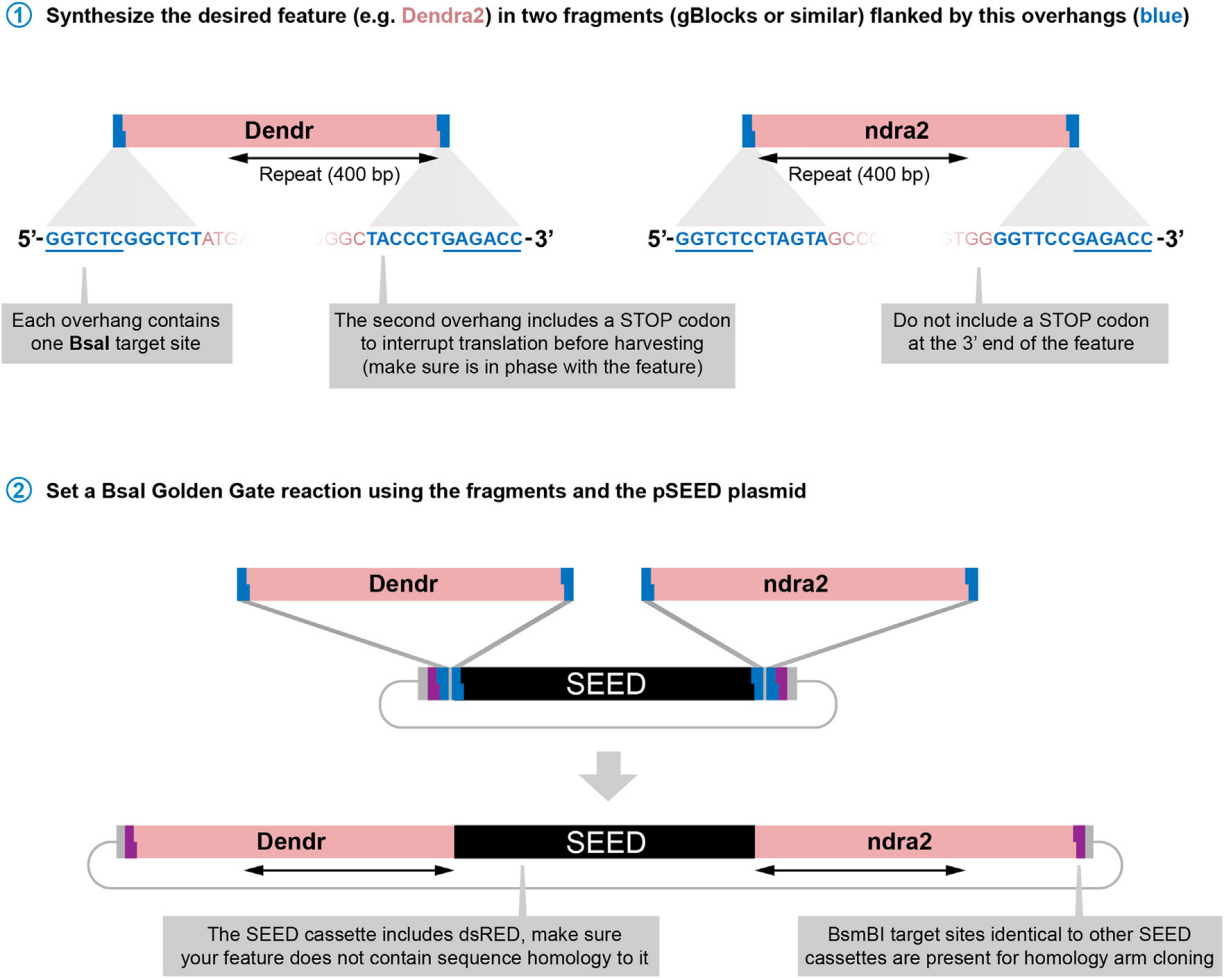
Generation of new SEED cassettes

In those occasions in which no SEED cassette is available for the chosen tag, a new SEED cassette can be easily generated by Golden Gate cloning following Figure 17. This cloning uses BsaI sites and produces a plasmid that is ready to clone using BsmBI. This cassette will be compatible with the homology arms used for the other SEED cassettes.

***Note:*** the pSEED-BsaI plasmid has Kan resistance.

## Expected outcomes

The expected outcome of this protocol is the generation of in-frame knockins that do not leave any genetic scar (except, in some occasions, silent mutations to avoid gRNA targeting). For most genes, the efficiency of this method is relatively high, for both the insertion (10%–75% of founders give rise to dsRED) and for the harvesting (we have obtained rates from 20%‒60% using 100 bp of homology and 75%–100% using 400 bp). Cloning using Golden Gate technology ensures fast and robust cloning. In our hands, cloning using these methods produces the correct insertion in most of the clones.

## Limitations

SEED/Harvest presents several limitations. The first one is the editing of highly repeated locus. While this is a general limitation of other current technologies, it is especially difficult for SEED/Harvest to editing these loci, as it depends on SSA for the harvesting step. Moreover, SEED/Harvest will need to be carefully adapted if small insertions are to be made (less than 100 bp). In these cases, SSA is likely not preferred over other DNA repair pathways during harvesting. In these cases, we recommend extending the repeated sequence to the endogenous locus and not only within the tag.

## Troubleshooting

For troubleshooting on the following topics, please see the following associated steps.

### Problem 1

Availability of gRNAs, please refer to the Notes for Step 2.

### Problem 2

Design and cloning of the donor vector, please refer to Notes for Steps 14‒17.

### Problem 3

Screening of Knockins, please refer to Notes for Step 26.

### Problem 4

Confirmation of precise insertion by PCR, please refer to Steps 30, 35 and 17.

### Problem 5

Harvesting of SEED alleles, please refer to Steps 38 and 39.

## Resource availability

### Lead contact

Further information and requests for resources and reagents should be directed to and will be fulfilled by the lead contact, Markus Affolter (markus.affolter@unibas.ch).

### Technical contact

For any technical information on the protocol, please contact the technical contact, Gustavo Aguilar (gusag@mit.edu).

### Materials availability

Plasmids are available in Addgene with the indicated numbers number (see key resources table) or under request to the lead author. Fly stocks are available in Bloomington under the indicated number (see key resources table) or under request to the lead author.

### Data and code availability

• No large-scale datasets have been generated in this study. The raw microscopy data that support the findings of this study are available from the lead author upon reasonable request.
• Any additional information required to reanalyze the data shown in this paper is available from the lead contact upon request
